# A facile route to old and new cyclophanes via self-assembly and capture

**DOI:** 10.1038/ncomms11052

**Published:** 2016-04-04

**Authors:** Mary S. Collins, Matthew E. Carnes, Bryan P. Nell, Lev N. Zakharov, Darren W. Johnson

**Affiliations:** 1 Department of Chemistry and Biochemistry and the Materials Science Institute, University of Oregon, Eugene, Oregon 97403-1253, USA; 2 Center for Advanced Materials Characterization in Oregon (CAMCOR), 1241 University of Oregon, Eugene, Oregon 97403-1241, USA

## Abstract

Cyclophanes are a venerable class of macrocyclic and/or cage compounds that often feature high strain, unusual conformations and quite surprising properties, many of which are legendary in physical organic chemistry. However, the discovery of new, diverse cyclophanes and derivatives has been hindered by syntheses that are traditionally low-yielding, requiring long reaction times, laborious purification steps and often extreme conditions. Herein, we demonstrate a new self-assembly route to a variety of discrete cyclic and caged disulfide structures, which can then be kinetically captured upon sulfur extrusion at room temperature to give a diversity of new thioether (hetera)cyclophanes in high yield. In addition to the synthesis of novel macrocycles (dimers through hexamers), this process provides an improved route to a known macrobicyclic trithiacyclophane. This technique also enables the facile isolation of a tetrahedral macrotricyclic tetrathiacyclophane in two steps at an ambient temperature.

## Abstract (web summary)

Cyclophanes—consisting of aromatic rings formed into a macrocycle—can display interesting properties but are challenging targets for synthesis. Here, the authors report a route to form cyclophanes by dynamic covalent chemistry, forming disulfides that can be subsequently converted to stable thioethers.

## Graphical abstract

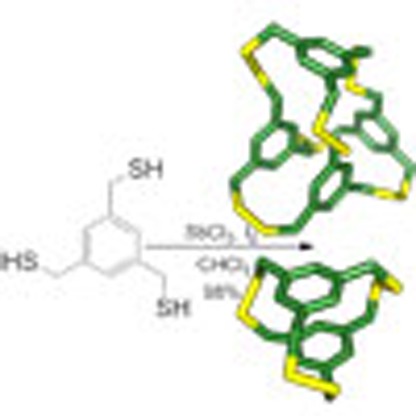

Since the first synthesis of anti-[2.2]metacyclophane by Pellegrin in 1899 (ref. 1) and [2.2]paracyclophane by Cram *et al*. in 1951 (ref. 2), there has been wide interest in the unusual properties of cyclophanes and related cage molecules. The confined geometries of the often distorted aromatic rings in these classes of molecules have allowed many studies of the fundamental properties of aromaticity itself^2^,^3^,^4^. The well-defined topology and high strain of cyclophanes has found utility in a number of applications including asymmetric catalysis^5^, insulating plastics^6^, organic electronics^7^, metal capture^8^,^9^ and most recognizably in the widely used parylene process in industry. Unfortunately, the field of cyclophane chemistry has been hindered by a lack of high yielding and functional group-tolerant preparative methods for their synthesis. A route to synthesize cyclophanes in a more efficient and selective way would be a substantial advancement to conventional cyclophane syntheses, and may bring forth new fundamental studies on their diverse properties as well as potentially advance applications as single-source precursors for insulating, conducting and dielectric polymers^10^. In this paper, we highlight a new method based on disulfide self-assembly followed by capture using a sulfur extrusion agent that enables high-yield syntheses of old and new macrocyclic cyclophanes, a macrobicyclic cyclophane and two new tetrahedral cyclophanes.

We recently showed self-assembly of a simple *p*-xylyl bridged dithiolate ligand, in the presence of AsCl_3_, rapidly forms a collection of discrete disulfide macrocycles upon treatment with iodine, rather than the disulfide oligomers/polymers that dominate under standard reaction conditions in the absence of AsCl_3_ (ref. 11)

While studying this dynamic behaviour in an oxidizing environment, we now reveal the role of the external pnictogen (in this case, SbCl_3_) to be more powerful than initially surmised. The directing behaviour of the SbCl_3_ additive exhibits impressive resistance to insoluble disulfide polymer formation, long reactions times and low selectivity for a variety of different ligand systems. Using this self-assembly method, the isolation of previously unattainable disulfide cyclophanes—including trimers, tetramers, pentamers, hexamers and tetrahedra—is now possible in a nearly instantaneous reaction from simple di- and tri-thiol ligands. Furthermore, from the cyclic and polycyclic disulfides, kinetic capture by desulfurization using a phosphoramide reagent provides a direct route to thiacyclophanes, again in exceptionally high yield. Herein we detail applications of our new method to the synthesis of a series of both known and entirely new cyclophanes.

## Results

### Facile synthesis of macrocyclic thioether cyclophanes

Previous methods to synthesize cyclophanes have relied on intra- and intermolecular macrocyclizations at high dilution and extreme temperatures. In many cases, indiscriminant homocouplings such as Wurtz or McMurry couplings^1^,^12^,^13^,^14^,^15^ are used where a mixture of oligomers and polymer is the primary product. In some more selective cases, Wittig reactions^16^,^17^ can be used to make mixed-macrocycles, but these instances still rely on unfavourable ring formation steps. Despite the significant breadth of cyclophane applications, their synthesis is still heavily confined to synthetic methods devised more than 40 years ago. These processes, although often spectacularly successful^18^, provide limited selectivity, low yields and require difficult purifications. Retrosynthetically, we imagined a different strategy to make these molecules in which the dynamic covalent chemistry of disulfides could be used to first form discrete disulfide assemblies followed by sulfur extrusion. In this way, the usually low yielding cyclization step is replaced by a self-assembly performed under thermodynamic control in the presence of a pnictogen trichloride additive. The disulfide macrocycles can then be treated with phosphoramides to remove sulfur atoms to contract the large ring resulting in heteracyclophane macrocycles (**1**, **2**) or larger species (*vide infra*).

Seminal work by Otto, Furlan and Sanders established that libraries of disulfide macrocycles can respond to external stimuli (such as a guest) to amplify specific components of equilibrating ‘combinatorial' mixtures^19^,^20^,^21^,^22^: the dynamic covalent self-assembly of thiols has emerged as an active area of investigation^23^,^24^,^25^,^26^,^27^. Our method allows a route to tune the composition of such libraries to form specific, discrete assemblies that can then be isolated using recirculating gel permeation chromatography or crystallization, and ultimately captured by sulfur extrusion to provide a remarkable array of kinetically stable, diverse two-dimensional and three-dimensional (3D) cyclophanes (see experimental details in Supplementary Information). The use of a pnictogen trichloride additive (for example, SbCl_3_) is critical during the self-assembly step to encourage the formation of discrete disulfide species, perhaps serving an organizational role in bringing together multiple thiolates to form a discrete assembly through a combination of Sb-S and Sb-π interactions^28^,^29^,^30^,^31^,^32^,^33^,^34^. This two-step self-assembly and capture process appears to be general and occurs in high yield.

**Figure 1:**
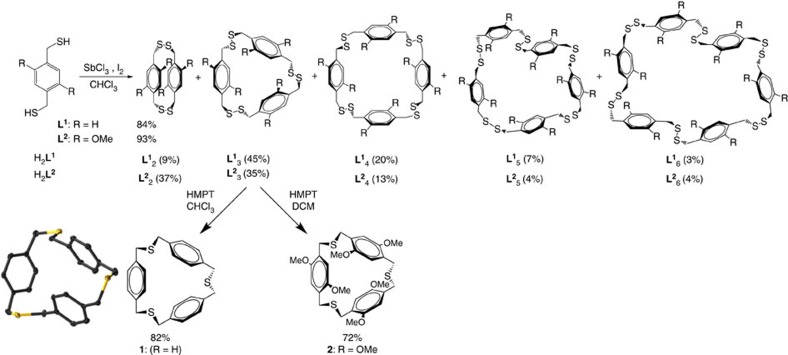
Synthesis and crystal structure of disulfide and thioether paracyclophanes. Pnictogen-activated iodine oxidation of H_2_**L**^**1**^ and H_2_**L**^**2**^ to form disulfide macrocycles (∼1 equiv ligand, 0.5 equiv SbCl_3_; 2 equiv I_2_): **L**^**1,2**^_2_ (dimer), **L**^**1,2**^_3_ (trimer) and **L**^**1,2**^_4_ (tetramer); (**L**^**1,2**^_5_ pentamers and **L**^**1,2**^_6_ hexamers are not shown, see single crystal structures of **L**^**1**^_**5**_ and **L**^**1**^_**6**_ in Supplementary Fig. 1). Overall isolated yields for the self-assembly are 93% and 84%, respectively for H_2_**L**^**1**^ and H_2_**L**^**2**^. The distribution of each disulfide macrocycle formed at ∼2 mM reaction conditions are shown. There is no evidence of oligomer/polymer formation by NMR or post GPC purification. Desulfurization of disulfide trimers yields thioether macrocycles **1** and **2** (single crystal X-ray structure representations of **1** shown with thermal ellipsoids at 50%. Hydrogen atoms and solvents of crystallization have been omitted for clarity).

As an example of a facile self-assembly and capture, we turned to constructing thiacyclophane **1** (Fig. 1), which is a challenging target by classical coupling methods. From [**L**^**1**^_3_], desulfurization with HMPT (hexamethylphosphorous triamide) in chloroform provides **1** in 82% isolated yield after 3 h and quantitative conversion after 6 days by ^1^H-NMR spectroscopy. The ^1^H NMR and ^13^C NMR spectra are simple, exhibiting time-averaged symmetry in solution. The ^1^H NMR of **1** shows only two signals, corresponding to the aromatic and benzylic protons, while the ^13^C spectrum shows three peaks arising from the three carbon environments. The desulfurization of unsubstituted **1** would not go to completion in dichloromethane or in the presence of a weaker sulfur extrusion agent, HEPT (hexaethylphosphorous triamide). The crude product, an iridescent white solid, was purified by trituration with water to remove any residual phosphine sulfide and separated using centrifugation. This purification method was found to give the highest isolated yield, serving as an optimal purification technique over separatory funnel extraction. Crystals suitable for X-ray diffraction were grown by vapour diffusion of pentane into chloroform or by slow evaporation of the chloroform solution directly (Fig. 1, bottom). **1** crystallizes in space group *P*2_1_/*c* without any additional solvent of co-crystallization. In addition, the arene rings in **1** are not coplanar, but rather adopt a face-to-face orientation in the solid state. This is commonly seen in paracyclophanes^35^ presumably to relieve strain, in which the arenes orient perpendicular to the macrocycle plane and the methylene protons are located along the macrocyclic equator. The central cavity of **1** has a diameter of 9 Å lengthwise. One of the thioether bond angles is slightly acute (C–S–C angles: 90.69°, 98.58° and 103.55°; typical thiother bond angles are 100–105°; ref. 36) and the average ring distance between the two closest benzene rings is 3.968 Å, too great a distance to suggest any attractive intramolecular aromatic interactions. The unsubstituted trimer **1** is robust in solution and does not appear to decompose with long-term exposure to oxygen, light or silica.

Given the facile conversion of the dithiol ligand H_2_**L**^**1**^ to the thioether trimer **1** in two steps, we attempted a one-pot reaction to directly transform H_2_**L**^**1**^ into trithioether **1** upon treatment with SbCl_3_, I_2_ and phosphoramide. Remarkably, crude ^1^H-NMR spectra and mass spectrometry data indicate successful one-pot conversion without further optimization of the reaction conditions (Supplementary Figs 12 and 14). It should be emphasized that only in very rare cases are cyclic trimers favoured during traditional cyclophane syntheses and in such cases self-assembly in a single pot is certainly not possible. The ease with which disulfide [**L**^**1**^_3_] forms during oxidation and then undergoes desulfurization to give trithioether **1** was quite unexpected, with an unoptimized overall two-step yield >50%.

Initial studies also suggest this route exhibits methoxy-functional group tolerance. As a preliminary test, iodine oxidation of 2,5-dimethoxy-1,4-bis(thiomethyl)benzene (H_2_**L**^**2**^) using SbCl_3_ similarly undergoes selective oxidation to produce [**L**^**2**^_2_] (dimer), [**L**^**2**^_3_] (trimer), [**L**^**2**^_4_] (tetramer), [**L**^**2**^_5_] (pentamer) and [**L**^**2**^_6_] (hexamer) in a combined 93% isolated yield (Fig. 1). The ^1^H NMR spectra of the structures are again quite simple, indicative of high symmetry molecules (Fig. 1 and Supplementary Fig. 2). The aromatic protons exhibit an upfield shift as the macrocycle decreases in size, presumably due to shielding effects by benzene rings that are oriented ‘face-to-face'^35^. Unlike the unsubstituted adducts, the formation of higher ordered [**L**^**2**^] disulfide-based cyclophanes (that is, tetramer, pentamer, hexamer and so on) require longer reaction times. For example, the intermolecular conversion to larger cyclic disulfides increases after 16–24 h at an ambient temperature. (In addition, this slow conversion to higher ordered cyclophanes, like trimers, tetramers, pentamers and hexamers, can be mediated by the amount of SbCl_3_, concentration and choice of solvent). Similar to the sulfur extrusion that produces **1**, thiacyclophane **2** forms in 72% yield by desulfurization of [**L**^**2**^_3_] using HMPT in 4 h in dichloromethane or 2 h in chloroform (Fig. 1).

### Macrobicyclic and tetrahedral cyclophanes

**Figure 2:**
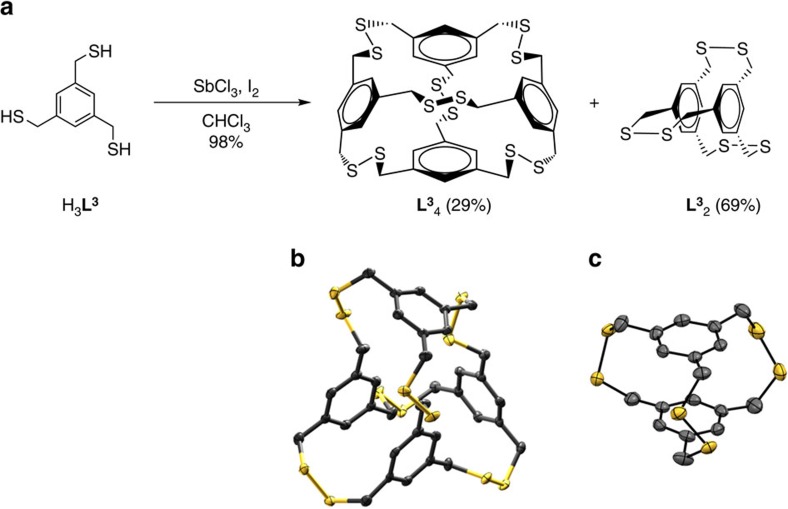
Synthesis and crystal structure of disulfide dimer and tetrahedron. (**a**) Pnictogen-activated iodine oxidation of H_3_**L**^**3**^ cleanly forms disulfide macrobicycle **L**^**3**^_2_ (dimer, 69%) and tetrahedron **L**^**3**^_4_ (tetramer, 29%) in combined 98% isolated yield at ∼2 mM concentration (1 equiv H_3_**L**^**3**^, 2.5 equiv SbCl_3_; 3 equiv I_2_). Single-crystal X-ray structure representations of tetrahedron **L**^**3**^_4_ (**b**) (thermal ellipsoids at 50%) and dimer **L**^**3**^_2_ (**c**) (thermal ellipsoids at 30%). Hydrogen atoms and solvents of crystallization have been omitted for clarity.

The preparation of highly bridged, 3D cyclophanes has suffered from lengthy and tedious syntheses requiring stepwise modification and addition of each bridging component^37^. Larger capsule-like molecules with similar structural features such as cubes and tetrahedra have been synthesized^2^,^8^,^38^. Despite numerous exotic and spectacular 3D cyclophanes, limitations in the classical stepwise syntheses have impeded efforts to design and prepare new, large cyclophane structures. Using our self-assembly and capture strategy, we used three-fold symmetric ligands in an attempt to synthesize higher ordered cyclophane structures such as tetrahedra. Pnictogen-activated oxidation of a trisubstituted thiol ligand (H_3_**L**^**3**^) with iodine gives a clean distribution of previously described disulfide dimer [**L**^**3**^_2_] as well as a distorted tetrahedron [**L**^**3**^_4_] in a combined 98% isolated yield after purification (Fig. 2). These species were initially identified by diffusion-ordered ^1^H-NMR spectroscopy where the diffusion coefficients for the dimer and tetrahedron differ (Supplementary Fig. 15). Control reactions using only iodine and H_3_**L**^**3**^ revealed that the pnictogen trichloride is required for fast, selective conversion to discrete **L**^**3**^ disulfides (Supplementary Figs 23 and 24).

The dynamic covalent behaviour driving the distribution of products during self-assembly appears largely influenced by three components: (1) concentration, (2) solvent effects and (3) reaction time. For example, the presence of SbCl_3_ influences the [**L**^**3**^_2_]:[**L**^**3**^_4_] ratio and also encourages immediate formation of both [**L**^**3**^_2_] and [**L**^**3**^_4_], which form sluggishly in the absence of the pnictogen as polymer formation competes (Supplementary Figs 23 and 24). In addition, the selectivity at low concentration shows [**L**^**3**^_2_] forms preferentially, whereas at high concentration, the tetramer [**L**^**3**^_4_] becomes more favoured (Supplementary Fig. 17). The solvent effects are also observed for the H_3_**L**^**3**^ pnictogen-assisted iodine oxidation. Reactions performed in dichloromethane result in a lowered isolated yield due to the formation of intermediate precipitates, while reactions in chloroform reveal no such precipitation. Last, formation of the tetrahedron is enhanced and can even surpass dimer formation using longer reaction times in a different solvent system, such as benzene (Supplementary Fig. 18). Full details of the scope and limitations of these variables and reaction conditions are currently being investigated and will be reported in a follow-up paper.

### Structure of dimer L^3^_2_

The disulfide dimer, **L**^**3**^_2_, was crystallized by slow evaporation of chloroform. The dimer crystallizes with *C*_2_ symmetry with two symmetrically independent molecules. The molecules are linked in the crystal structure via short intermolecular S...S close contacts. **L**^**3**^_2_ does not co-crystallize with solvent. The C–S–S–C torsion angles in each independent dimer deviate substantially from ideality (90°), adopting highly strained conformations (115.6°, 107.1°, 107.1°) and (120.0°, 120.0°, 116.3°). To better understand the prevalence for similar existing dihedral disulfide bond angles, a systematic survey of the Cambridge Structural Database (CSD) for disulfide bonds at 120°±5 was performed (Supplementary Fig. 19). Only 16 structures were identified with torsional angles at nearly 120°; most of them appeared as bridging units in cyclic structures. The rarity of such apparent strain in the disulfide bridges also mirrors the ‘stress' exhibited by the ligand seen by the deviation of the methylene carbon from the benzene plane (see experimental details in Supplementary Information). The C6-rings in both molecules are planar within 0.006 and 0.007 Å, but the methylene atoms are significantly ‘puckered' out of the C6-ring plane: 0.12(0.1) −0.19(0.1) Å. This is consistent with early work by Houk and Whitesides^39^, who suggested that tris-disulfide **L**^**3**^_2_ does not favourably form upon oxidation: ‘only polymeric disulfide products were obtained…molecular models suggest that…[**L**^**3**^_2_ is]…seriously strained…[so] it is not surprising that these substances do not form', again indicating the powerful role the pnictogen trichloride plays in the self-assembly process.

**Figure 3:**
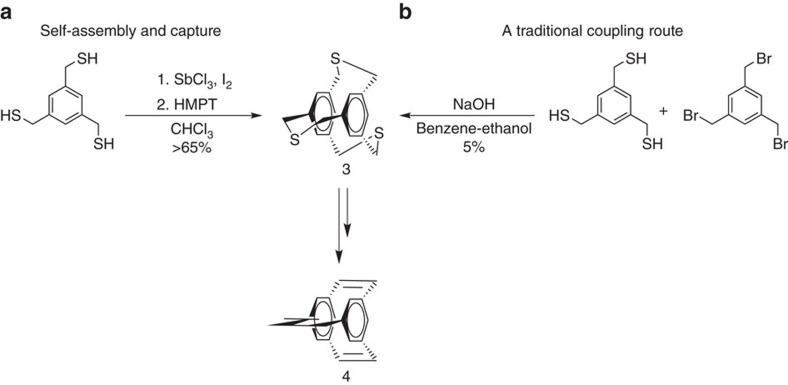
Self-assembly and capture of a known cyclophane. (**a**) Self-assembly of H_3_**L**^**3**^ (1 mol SbCl_3_, 1.2 mol I_2_; 69%) followed by capture (95%) by sulfur extrusion with HMPT (4 mol) provides known trithiacyclophane **3** in overall 65% yield. (**b**) A traditional stepwise route to **3** proceeds in 5% yield.

To showcase the utility of the sulfur extrusion process, we next sought to prepare the known 2,11,20-trithia[3.3.3](1,3,5)cyclophane^13^,^40^,^41^ **3** by exposing tris(disulfide) [4.4.4](1,3,5)cyclophane [**L**^**3**^_2_] to a stoichiometric amount of HMPT for 1.5 h at an ambient temperature. The trithiacyclophane **3** was formed in quantitative yield by ^1^H-NMR spectroscopy and isolated in 95% yield from disulfide [**L**^**3**^_2_] (Fig. 3, Supplementary Fig. 8). Over two steps, this provides an isolated yield of 65%, a significant improvement over the reported 5.3% yield^13^ observed by treatment of 1,3,5-tris(bromomethyl)benzene with 1,3,5-tris(mercaptomethyl)benzene H_3_**L**^**3**^ or the 24% optimized yield from 1,3,5-tris(bromomethyl)benzene and Na_2_S (ref. 41). Surprisingly, no evidence of ring opened, oligomeric thiother products has been observed using our route (Supplementary Figs 15, 16 and 21). Remarkably, this entire two-step self-assembly and capture method can be performed in one pot: treatment of H_3_**L**^**3**^ with SbCl_3_, I_2_ and HMPT also forms **3** as observed by ^1^H-NMR spectroscopy (Supplementary Fig. 11). Thioether **3** is known to serve as a starting point to form cyclophane **4** from established procedures^41^.

### Tetrahedral cages of hexadisulfide L^3^_4_ and hexathioether 5

**Figure 4:**
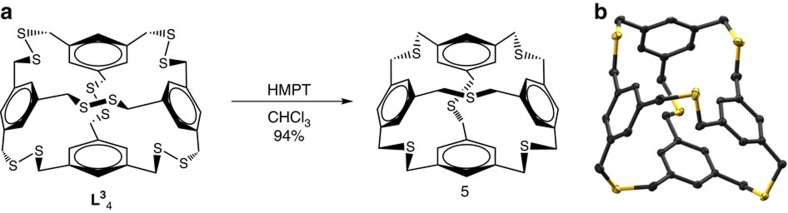
Synthesis and crystal structure of a thiatetrohedrophane. Desulfurization (**a**) of **L**^**3**^_4_ gives tetrathioether [3_4_]tetrahedrophane **5** (6 mol HMPT). Single-crystal X-ray structure representation of thiatetrahedrophane **5** (**b**); all thermal ellipsoids at 50%. Hydrogen atoms and solvents of crystallization have been omitted for clarity.

Encouraged by the success of sulfur extrusion on the trithioether dimer, we sought to perform sulfur extrusion on the hexadisulfide tetrahedron **L**^**3**^_4_. This would require the extrusion of six sulfur atoms (one on each edge) of the distorted tetrahedron—representing a total of 24 bonds broken/formed in this single step—to proceed with no polymerization and in high yield for each extrusion. We were delighted to discover that sulfur extrusion of all six disulfides to form a tetrahedral hexathioether ‘tetrahedrophane' **5**, proceeds by desulfurization with HMPT at an ambient temperature in 4 h in 94% yield (Fig. 4)^42^,^43^,^44^. To the best of our knowledge, this hexathioether assembly is unknown; for related concave spheriphanes, our method reduces an eight-step synthesis to two steps^45^. The formation of hexathioether **5** was confirmed by ^1^H NMR spectroscopy, exhibiting time-averaged tetrahedral symmetry in CDCl_3_ (Supplementary Figs 9 and 10), and single-crystal X-ray diffraction (Fig. 4b). The loss of six sulfur atoms decreases the size of the cyclic structure only slightly and the aromatic and methylene singlets display small, yet expected upfield shifts relative to the disulfide analogue in CDCl_3_.

Diffraction-quality single crystals of each tetrahedron **L**^**3**^_4_ and **5** were grown by vapour diffusion of pentane into chloroform or solvent evaporation. Both **L**^**3**^_4_ and **5** capsules exhibit robust chemical stability and do not show decomposition over time or with exposure to light. **L**^**3**^_4_ crystallizes in the space group *P*2_1_/*c* and surprisingly does not crystallize with a solvent guest inside its cavity. Instead, **L**^**3**^_4_ ‘folds in' on itself to avoid the formation of a large void in the solid state (Fig. 2b). Owing to this conformation, **L**^**3**^_4_ displays a wide range of non-ideal C-S-S-C torsional angles (82.93°, 85.61°, 88.46°, 88.80°, 92.62°, 113.24°). Hexathioether **5** crystallizes in the *P*2_1_/*n* space group and co-crystallizes with chloroform molecules of solvation, although its small cavity also remains empty. Like **L**^**3**^_4_, the thiatetrahedrophane **5** collapses in on itself avoiding unfavourable void space. The C–S–C angles within the cyclophane were in the expected range for cyclic thioethers (98.84°, 99.21°, 100.17°, 102.71°, 102.90°, 102.92°; ref. 36) In addition, the ‘folded in' conformation may also enable a slightly favourable aromatic interaction between two adjacent benzene rings, which reveal a centroid-to-centroid distance of 3.7 Å (Fig. 4b).

## Discussion

We have shown a selective, high yielding alternative to traditional cyclophane synthesis that uses self-assembly and capture through the use of a pnictogen additive to direct the self-assembly of discrete disulfide (hetera)cyclophanes. The book *Modern Cyclophane Chemistry* cites a need for simpler reaction pathways or procedures for preparing cyclophanes in larger quantities^46^. Using a self-assembly approach eliminates the need for caustic, high-temperature methods and avoids low yields that result from the rapid formation of unfavourable insoluble oligomers. This method complements modern research on dynamic combinatorial covalent libraries of disulfides^19^,^23^ by providing a method to isolate and kinetically capture individual members of such equilibrating systems; in addition, the key pnictogen additive during the self-assembly step avoids the formation of disulfide polymers that plagued early efforts aimed at synthesizing discrete polydisulfides^39^,^47^.

Rather than relying on traditional kinetic syntheses, this route relies on self-assembly to prepare complex cyclophanes as the major product of the reaction. This dynamic covalent method allows the synthesis of formerly inaccessible, higher ordered species such as trimers, tetramers, pentamers, hexamers, macrobicycles and tetrahedra to be performed cleanly and in high yield, and preliminary results indicate that this method is also tolerant to a simple functional group that enhances the solubility in polar solvents. Importantly, the self-assembly reaction is under thermodynamic control. For instance, treatment of ligand H_2_**L**^**1**^ with SbCl_3_ and I_2_ provides the same distribution of macrocyclic disulfides as does reaction of tetrameric disulfide [**L**^**1**^_**4**_] under the same conditions (see stacked NMR plot comparing the two reactions in Supplementary Fig. 25). These disulfide complexes operate as excellent precursors to their thioether analogues by treatment with sulfur extrusion agents, and such thioethers are known to be effective precursors to hydrocarbon cyclophanes, suggesting that this new synthetic method could open the door to the synthesis of myriad new hydrocarbon cyclophanes as well. In conclusion, our supramolecular approach serves as a powerful strategy in which new cyclophanes can be made in cases where strain and complex design are difficult to conceive or do not possess selectivity to be formed via classical coupling techniques. This strategy could be of particular interest to the future synthetic design of macrocycles and cage compounds, as well as toward the myriad applications for which cyclophanes are utilized, such as polymer-based materials (for example, the parylene process)^10^, catalysis, strained systems and host–guest binding/separations science^48^,^49^. We are mindful of the fact that this synthetic method enables the formation of many difficult-to-synthesize cyclophanes that may be of interest as monomers for new polymers or for use directly in the widely used parylene process in industry.

## Methods

### Single-crystal X-ray diffraction

Full synthetic and characterization details are provided in the supporting information. Single-crystal X-ray diffraction intensities were collected at 100 K (**L**^**3**^_4_, **5** and **1**), 150 K (**L**^**1**^_6_), 173 K (**L**^**1**^_5_), 200 K (**L**^**2**^_2_) and 223(2) K (**L**^**3**^_2_) on a Bruker Apex2 CCD diffractometer using MoKα radiation, *λ*=0.71073 Å, (**5, 1, L**^**2**^_2_) and CuKα radiation, *λ*=1.54178 Å, (**L**^**3**^_4_**, L**^**3**^_2_**, L**^**1**^_6_ and **L**^**1**^_5_). The space groups were determined based on systematic absences. Absorption corrections were applied by SADABS^50^. The structures were solved by direct methods and Fourier techniques and refined on *F*^2^ using full-matrix least-squares procedures. All the non-H atoms were refined with anisotropic thermal parameters. The H atoms in all the structures were refined in calculated positions in a rigid group model. Two S–S bridges in **L**^**3**^_2_ are disordered over two positions in a ratio 0.53/0.47. The –S- bridges in **1** are disordered as well: the three strongest peaks on the residual density map of **1** (values are in the range 2.58–3.69 e Å^−3^) seem to be related to a second position of the S atom, but its contribution was not taken into account in the final refinement. The Flack parameter for **L**^**3**^_2_ is 0.08(5). The structure of **L**^**2**^_2_ was checked in centro- and non-centrosymmetrical space groups and was determined in non-centrosymmetrical space group *Pna*2_1_. The structure of **L**^**2**^_2_ is a racemic twin consisting of left and right molecules in the ratio 0.68/0.32 and having a pseudo-inversion centre. The crystals of **L**^**1**^_6_ and **L**^**1**^_5_ are very thin needles and even using a strong Incoatec Cu *IμS* source, X-ray diffraction data for these crystals were weak especially at high angles. The data for **L**^**1**^_6_ were collected only up to 2*θ*_max_=96°. The data for **L**^**1**^_5_ were collected up to 2*θ*_max_=135°, but only reflections with 2*θ* less than 100° were used in the refinement. As a result, values of *R*_int_ for both compounds are high. The final X-ray structures for **L**^**1**^_6_ and **L**^**1**^_5_ are not precisely determined, but they clearly confirm the structure and compositions of these compounds and are therefore also included in this paper. All the calculations were performed by the Bruker SHELXTL (v. 6.10; ref. 51) and SHELXL-2013 packages^52^.

### Crystallographic data for L^3^_4_

C_36_H_36_S_12_, *M*=853.37, 0.19 × 0.05 × 0.03 mm, *T*=100(2) K, monoclinic, space group *P*2_1_/*c*, *a*=18.6720(16) Å, *b*=12.2920(10) Å, *c*=16.8619(15) Å, *β*=94.755(6)°, *V*=3,856.8(6) Å^3^, *Z*=4, *D*_c_=1.470 Mg m^−3^, *μ*(Cu)= 6.524 mm^−1^, *F*(000)=1,776, 2*θ*_max_=130.0°, 22,999 reflections, 6,373 independent reflections(*R*_int_=0.0765), R1=0.0622, wR2=0.1623 and GOF=1.018 for 6,373 reflections (433 parameters) with *I*>2*σ*(*I*), R1=0.0762, wR2=0.1781 and GOF=1.018 for all the reflections, max/min residual electron density +0.893/−0.607 eÅ^−3^.

### Crystallographic data for 5

C_37_H_37_Cl_3_S_6_, *M*=780.38, 0.19 × 0.09 × 0.07 mm, *T*=100(2) K, monoclinic, space group *P*2_1_/*n*, *a*=8.8821(6) Å, *b*=16.2160(10) Å, *c*=25.2559(15) Å, *β*=91.188(1)°, *V*=3,636.9(4) Å^3^, *Z*=4, *D*_c_=1.425 Mg m^−3^, *μ*(Mo)=0.624 mm^−1^, *F*(000)=1,624, 2*θ*_max_=50.0°, 27,936 reflections, 6,404 independent reflections (*R*_int_=0.0419), R1=0.0436, wR2=0.0975 and GOF=1.029 for 6,404 reflections (447 parameters) with *I*>2*σ*(*I*), R1=0.0610, wR2=0.1070 and GOF=1.029 for all reflections, max/min residual electron density +1.673/−0.827 eÅ^−3^.

### Crystallographic data for 1

C_24_H_24_S_3_, *M*=408.61, 0.24 × 0.16 × 0.09 mm, *T*=100(2) K, monoclinic, space group *P*2_1_/*n*, *a*=18.4134(17) Å, *b*=5.7476(6) Å, *c*=20.3204(18) Å, *β*=107.774(4)°, *V*=2047.9(3) Å^3^, *Z*=4, *D*_c_=1.325 Mg m^−3^, *μ*(Mo)= 0.369 mm^−1^, *F*(000)=864, 2*θ*_max_=50.0°, 28,649 reflections, 3,596 independent reflections (*R*_int_=0.0443), R1=0.0982, wR2=0.2422 and GOF=1.134 for 3,596 reflections (244 parameters) with *I*>2*σ*(*I*), R1=0.1048, wR2=0.2474 and GOF=1.134 for all the reflections, max/min residual electron density +3.691/−0.0915 eÅ^−3^.

### Crystallographic data for L^3^_2_

C_18_H_18_S_6_, *M*=426.68, 0.09 × 0.06 × 0.04 mm, *T*=223(2) K, monoclinic, space group *C*2, *a*=7.6361(10) Å, *b*=17.165(2) Å, *c*=15.419(2) Å, *β*=103.756(9)°, *V*=1,963.1(5) Å^3^, *Z*=4, *D*_c_=1.444 Mg m^−3^, *μ*(Cu)= 6.408 mm^−1^, *F*(000)=888, 2*θ*_max_=135.97°, 8,461 reflections, 3,328 independent reflections (*R*_int_=0.0689), R1=0.0557, wR2=0.1397 and GOF=1.044 for 3,328 reflections (255 parameters) with *I*>2*σ*(*I*), R1=0.0688, wR2=0.1486 and GOF=1.044 for all the reflections, the Flack=0.08(5), max/min residual electron density +0.255/−0.190 eÅ^−3^.

### Crystallographic data for L^1^_6_

C_50_H_50_Cl_6_S_12_, *M*=1,248.32, 0.42 × 0.02 × 0.01 mm, *T*=150(2) K, monoclinic, space group *P*2_1_/*c*, *a*=17.667(3) Å, *b*=5.4025(9) Å, *c*=30.355(5) Å, *β*=102.055(10)°, *V*=2,833.4(8) Å^3^, *Z*=2, *D*_c_=1.463 Mg m^−3^, *μ*(Cu)= 7.172 mm^−1^, *F*(000)=1,288, 2*θ*_max_=95.76°, 8,328 reflections, 2,619 independent reflections (*R*_int_=0.1616), R1=0.0798, wR2=0.1875 and GOF=1.023 for 2,619 reflections (307 parameters) with *I*>2*σ*(*I*), R1=0.1661, wR2=0.2406 and GOF=1.023 for all the reflections, max/min residual electron density +1.186/−0.520 eÅ^−3^.

### Crystallographic data for L^1^_5_

C_42_H_42_Cl_6_S_10_, *M*=1,080.05, 0.04 × 0.01 × 0.01 mm, *T*=173(2) K, monoclinic, space group *P*2_1_/*c*, *a*=25.778(5) Å, *b*=5.4791(14) Å, *c*=34.978(7) Å, *β*=98.066(12)°, *V*=4,891.4(18) Å^3^, *Z*=4, *D*_c_=1.467 Mg m^−3^, *μ*(Cu)= 7.437 mm^−1^, *F*(000)=2,224, 2*θ*_max_=135.36°, 24,422 reflections, 4,172 independent reflections (*R*_int_=0.2503), R1=0.1185, wR2=0.2866 and GOF=1.026 for 4,172 reflections (523 parameters) with *I*>2*σ*(*I*), R1=0.2530, wR2=0.3756 and GOF=1.026 for all the reflections, max/min residual electron density +0.685/−0.589 eÅ^−3^.

### Crystallographic data for **L** ^**2**^_**2**_

C_20_H_24_O_4_S_4_, *M*=456.63, 0.20 × 0.12 × 0.07 mm, *T*=200(2) K, orthorhombic, space group *Pna2*_1_, *a*=14.437(3) Å, *b*=16.405(3) Å, *c*=9.0971(17) Å, *V*=2,154.5(7) Å^3^, *Z*=4, *D*_c_=1.408 Mg m^−3^, *μ*(Mo)= 0.465 mm^−1^, *F*(000)=960, 2*θ*_max_=50.0°, 17,105 reflections, 3,781 independent reflections (R_int_=0.0649), R1=0.0490, wR2=0.1029 and GOF=1.038 for 3,781 reflections (254 parameters) with *I*>2*σ*(*I*), R1=0.0711, wR2=0.1143 and GOF=1.039 for all the reflections, max/min residual electron density +0.300/−0.237 eÅ^−3^.

## Additional information

**Accession code:** CCDC 1403626-1403631; CCDC 1421037 contain the supplementary crystallographic data for this paper. These data can be obtained free of charge from The Cambridge Crystallographic Data Centre via www.ccdc.cam.ac.uk/data_request/cif.

**How to cite this article:** Collins, M. S. *et al*. A facile route to old and new cyclophanes via self-assembly and capture. *Nat. Commun.* 7:11052 doi: 10.1038/ncomms11052 (2016).

## Supplementary Material

Supplementary Supplementary Figures 1-25, Supplementary Discussion, Supplementary Methods and Supplementary References
