# Supplementary material for: A facile route to old and new cyclophanes via self-assembly and capture
Source: Nat Commun. 2016 Apr 4;7:11052. doi: 10.1038/ncomms11052 (PMC4821999; doi:10.1038/ncomms11052)
Supplement: Supplementary — Figures 1-25, Supplementary Discussion, Supplementary Methods and Supplementary References [file ncomms11052-s1.pdf]

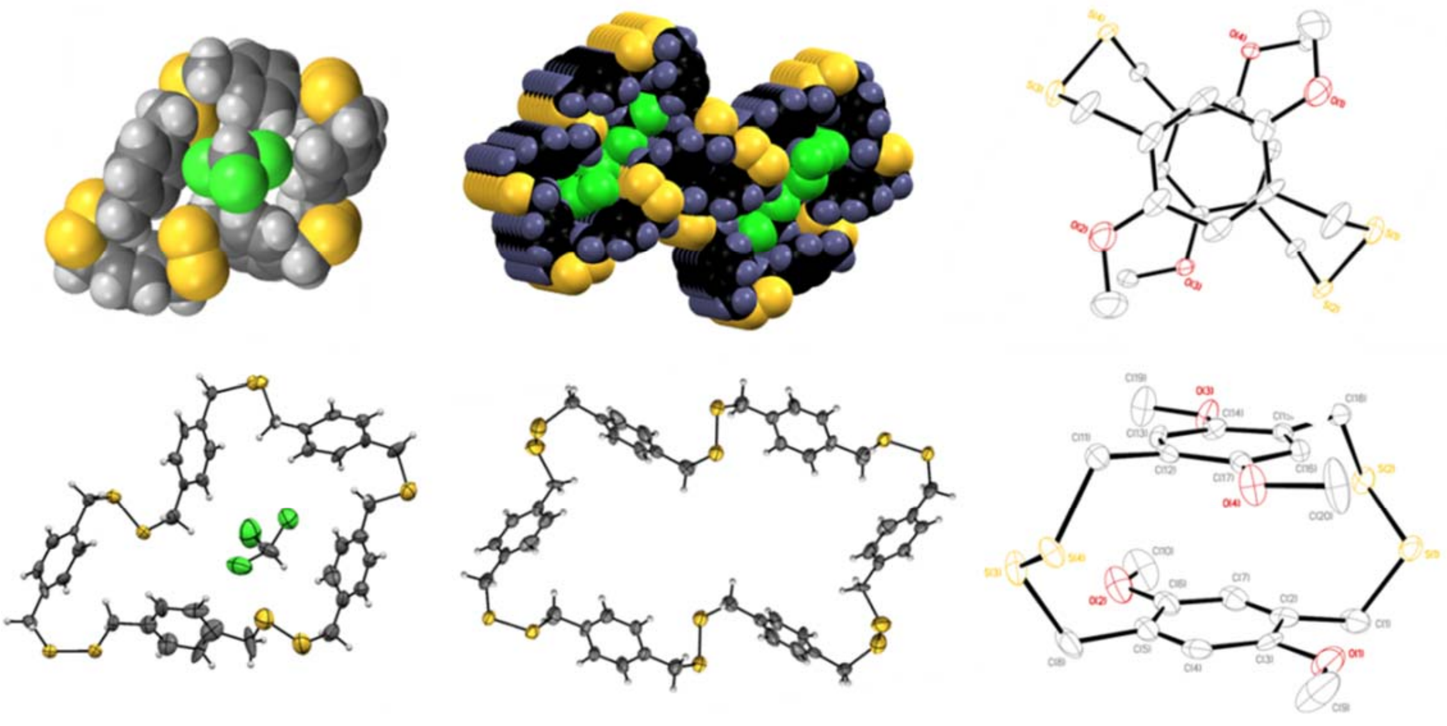

Supplementary Figure 1. Space-filling representation of $\mathbf{L^{1}}_{5}$ pentamer (top left) and $\mathbf{L^{1}}_{6}$ hexamer (top middle) and their single crystal X-ray structures (bottom left and middle). ORTEP diagram of $\mathbf{L^{2}}_{2}$ showing ellipsoids at 50% probability (top and bottom right).

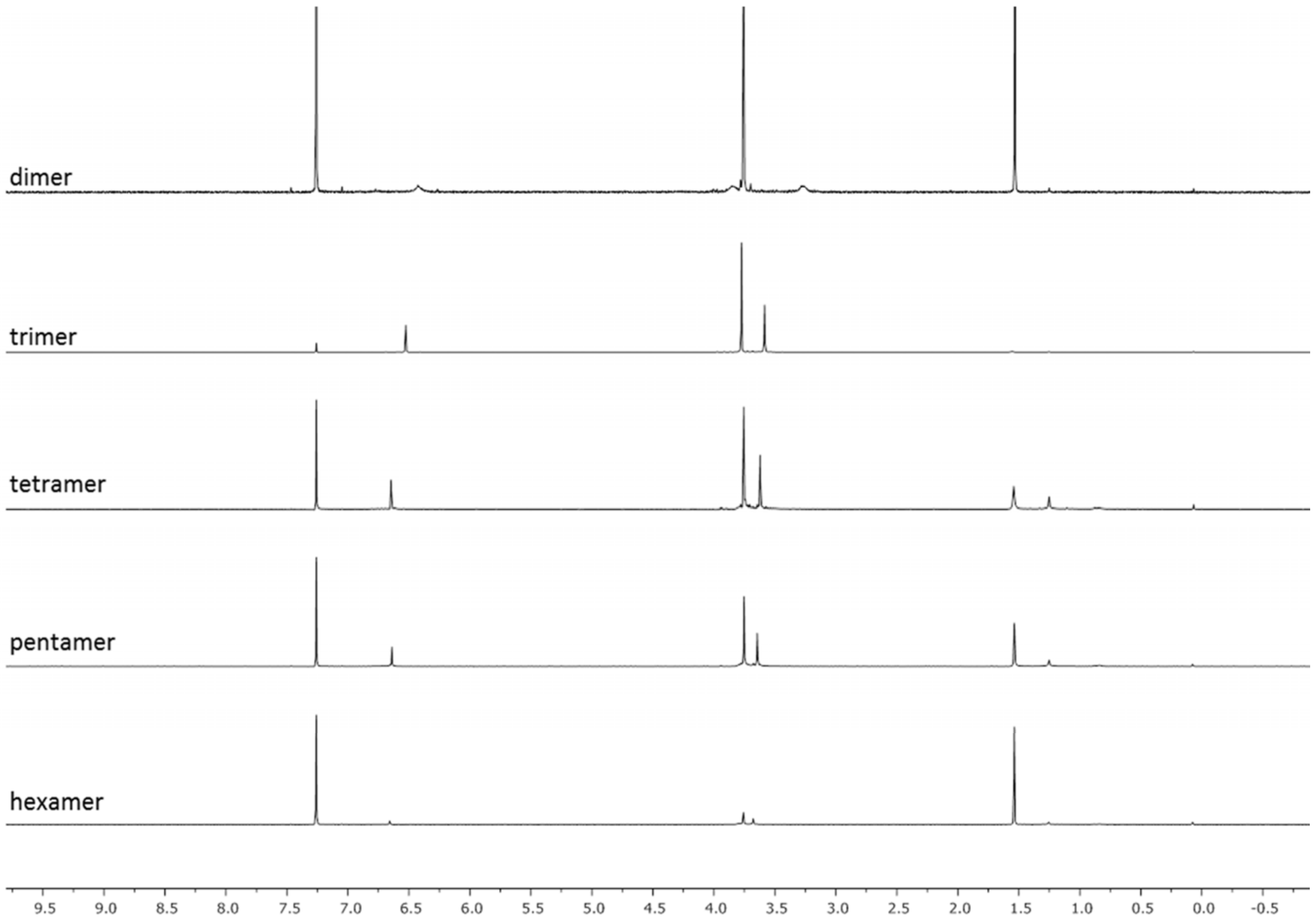

Supplementary Figure 2. ^{1}H NMR ($CDCl_3$ 300 MHz) of $\mathbf{L^2}_2$ (top), $\mathbf{L^2}_3$, $\mathbf{L^2}_4$, $\mathbf{L^2}_5$, and $\mathbf{L^2}_6$ (bottom) (H_2O – 1.5 ppm; H grease – 1.25, 0.84-0.87 ppm).

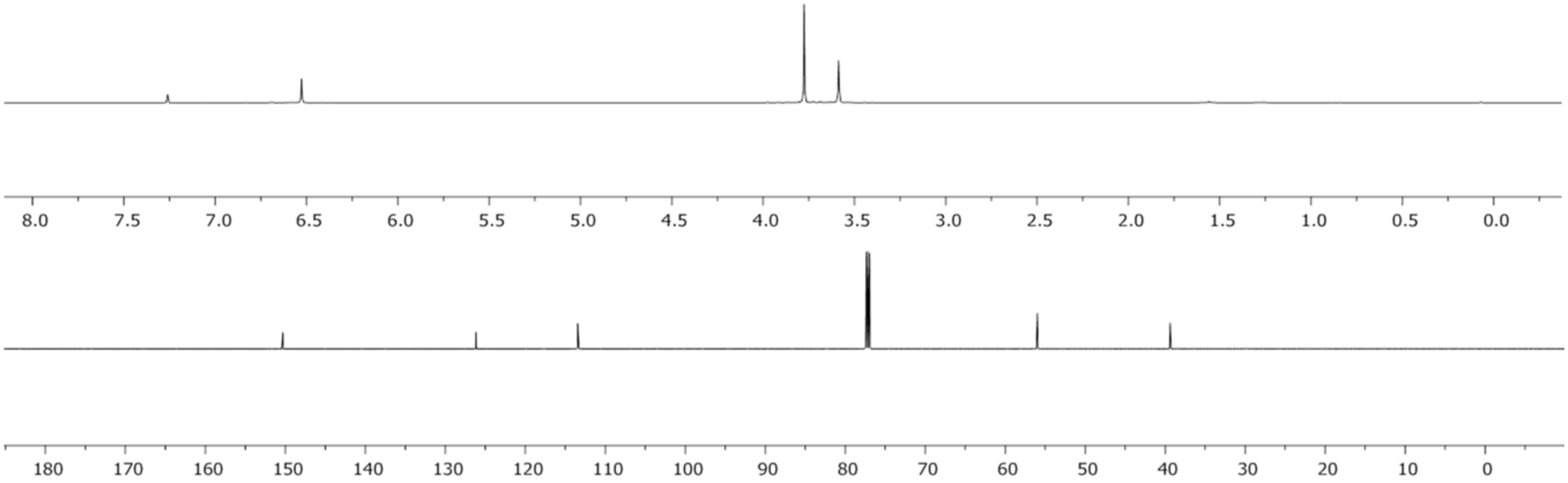

Supplementary Figure 3. ^{1}H NMR (top) and ^{13}C NMR (bottom) of **L^2**$_3$ in $CDCl_3$.

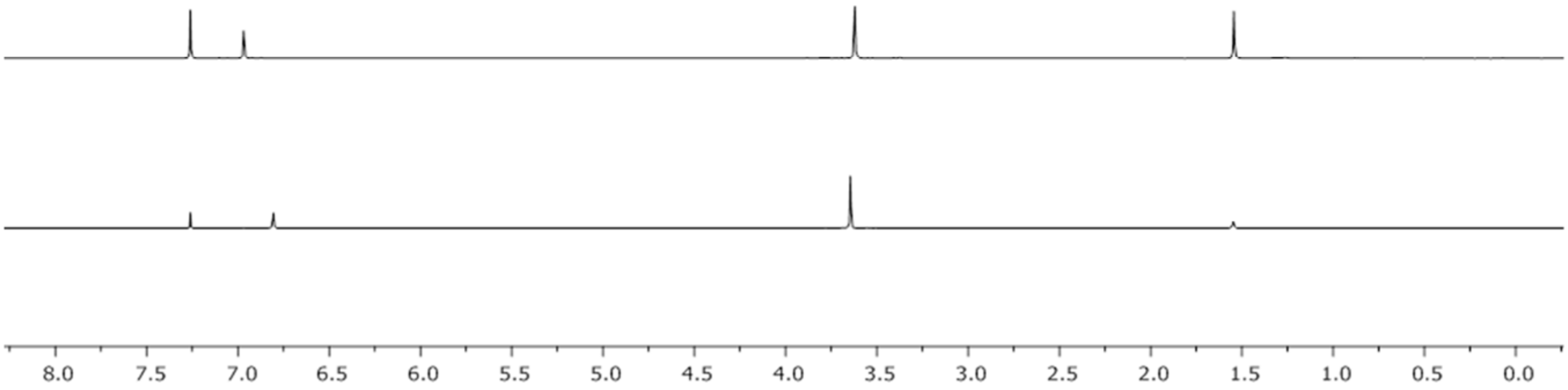

Supplementary Figure 4. ^{1}H NMR of **L³**$_4$ (top) and **L³**$_2$ (bottom) in $CDCl_3$.

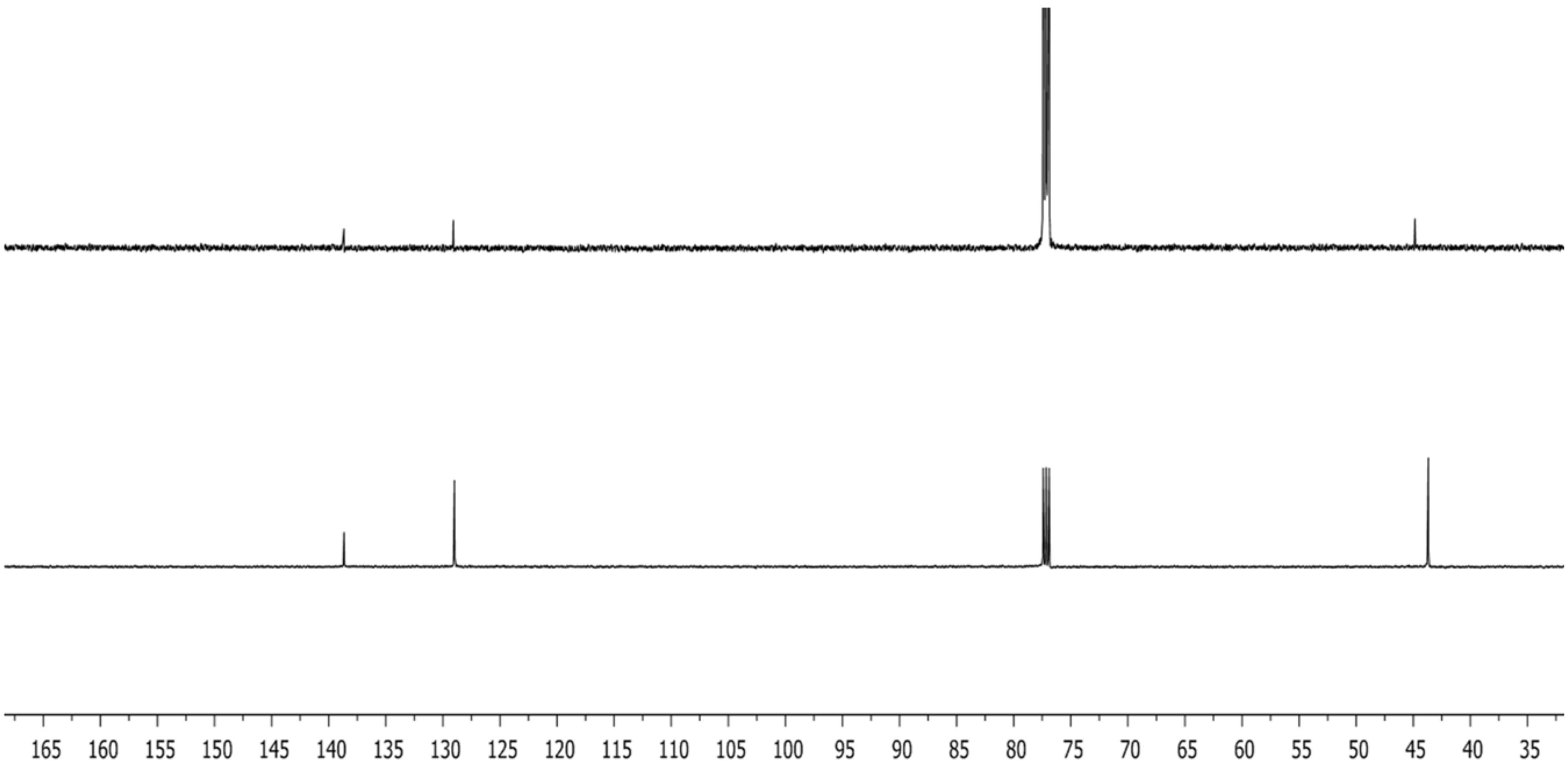

Supplementary Figure 5. ^{13}C NMR of $\mathbf{L^3}_4$ (top) and $\mathbf{L^3}_2$ (bottom) in $CDCl_3$.

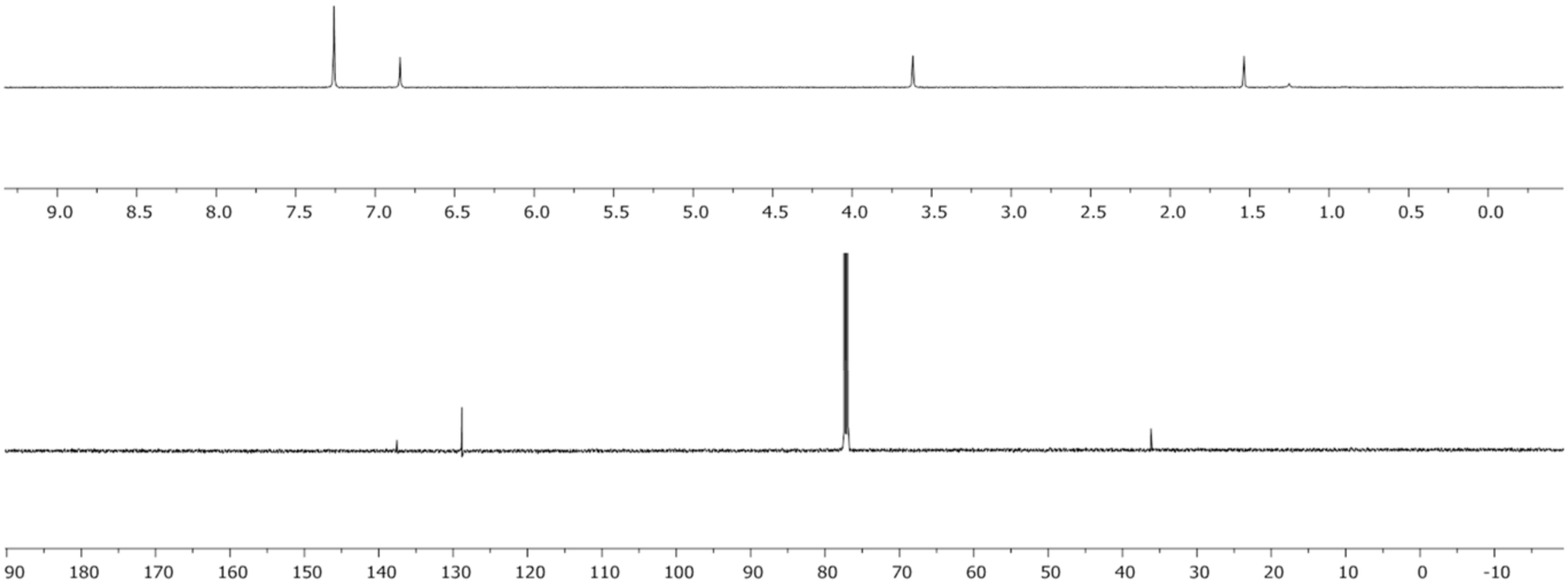

Supplementary Figure 6. ^{1}H NMR of **1** (top) and ^{13}C NMR of **1** (bottom) in $CDCl_3$.

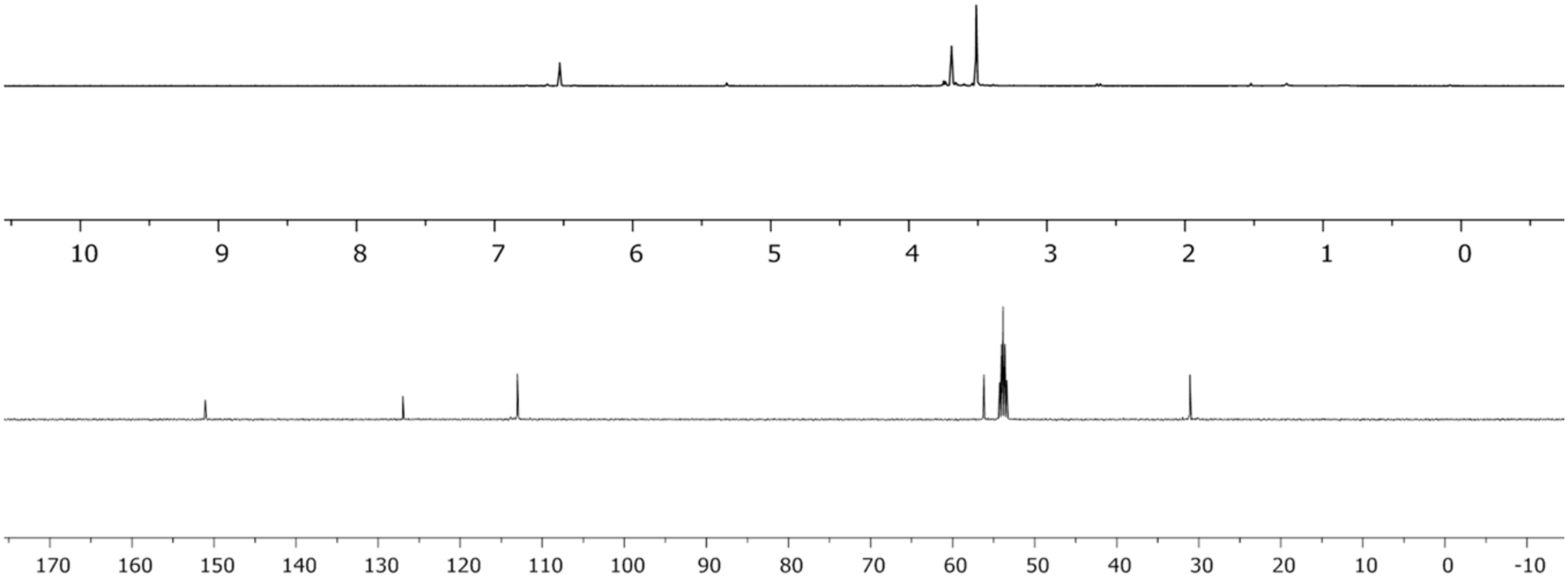

Supplementary Figure 7. ^{1}H NMR of **2** (top) and ^{13}C NMR of **2** (bottom) in CD_2Cl_2.

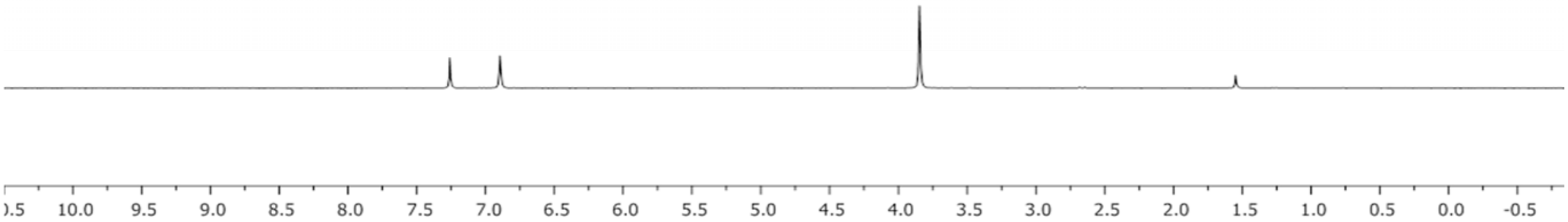

Supplementary Figure 8. ^1H NMR of **3** in $CDCl_3$.

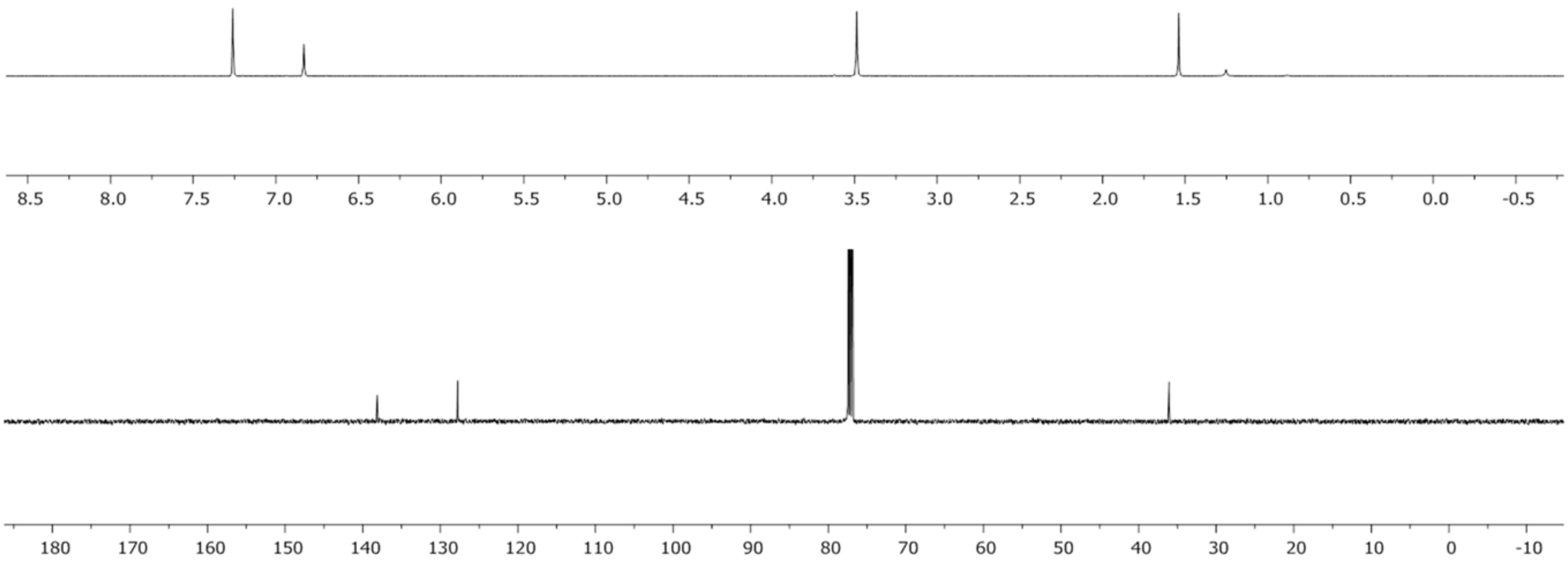

Supplementary Figure 9. ^{1}H NMR of **5** (top) and ^{13}C NMR of **5** (bottom) in $CDCl_3$.

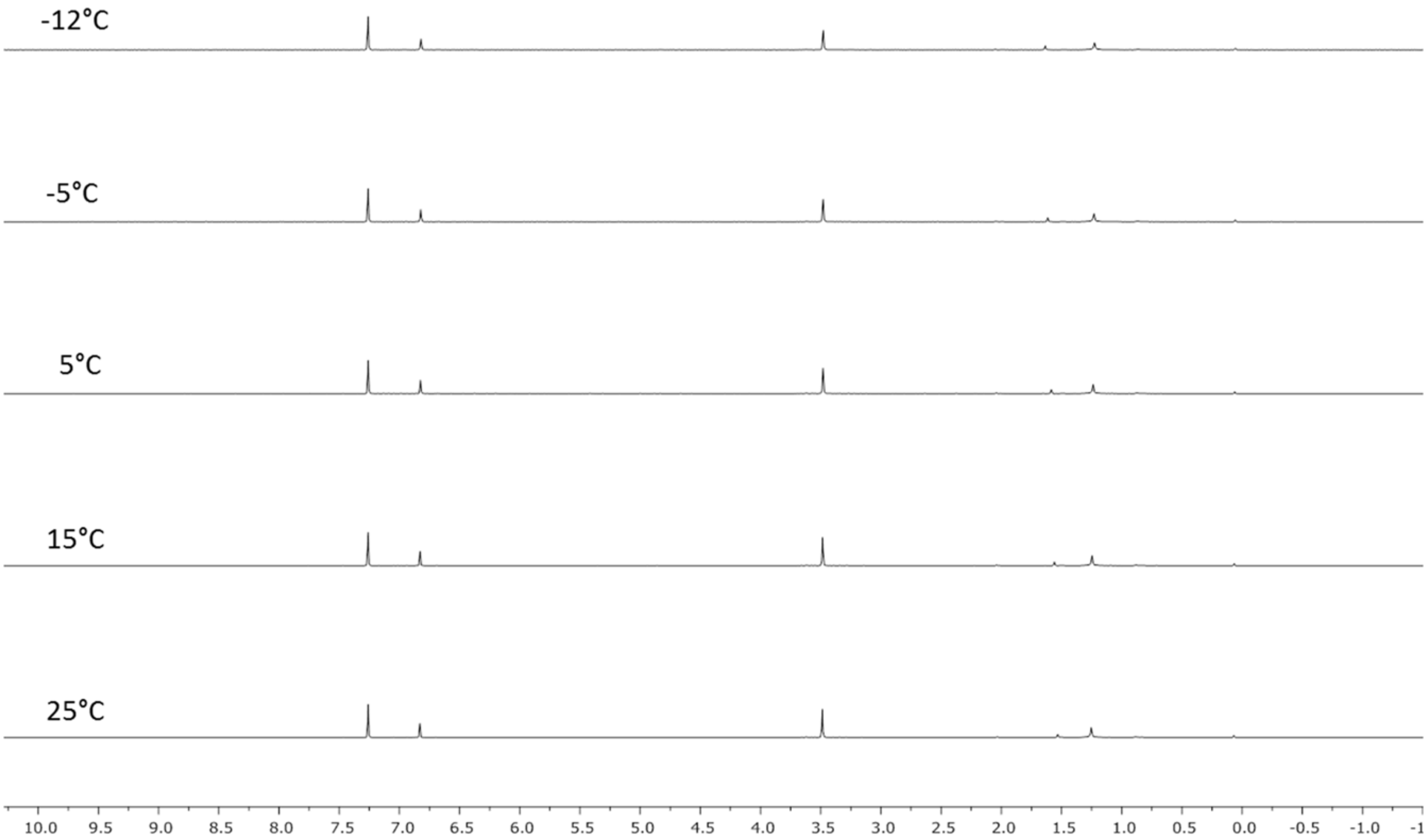

Supplementary Figure 10. VT-^{1}H NMR of **5** in $CDCl_3$ (H_2O – 1.5 ppm; H grease – 1.25, 0.84-0.87 ppm).

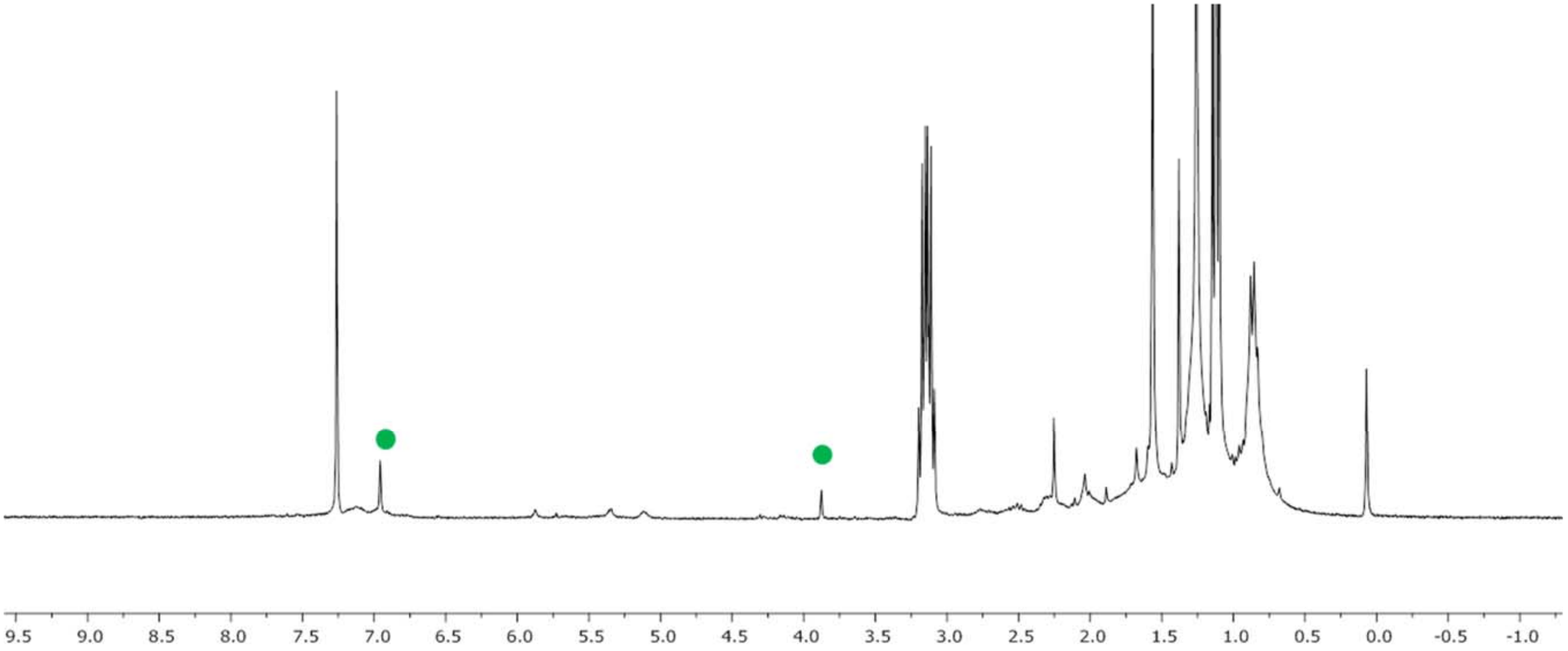

Supplementary Figure 11. ^{1}H NMR of crude mixture of one-pot reaction of H_3**L**^{3} with $SbCl_3$, I_2 and phosphoramide in $CDCl_3$. **3** is indicated by green circles.

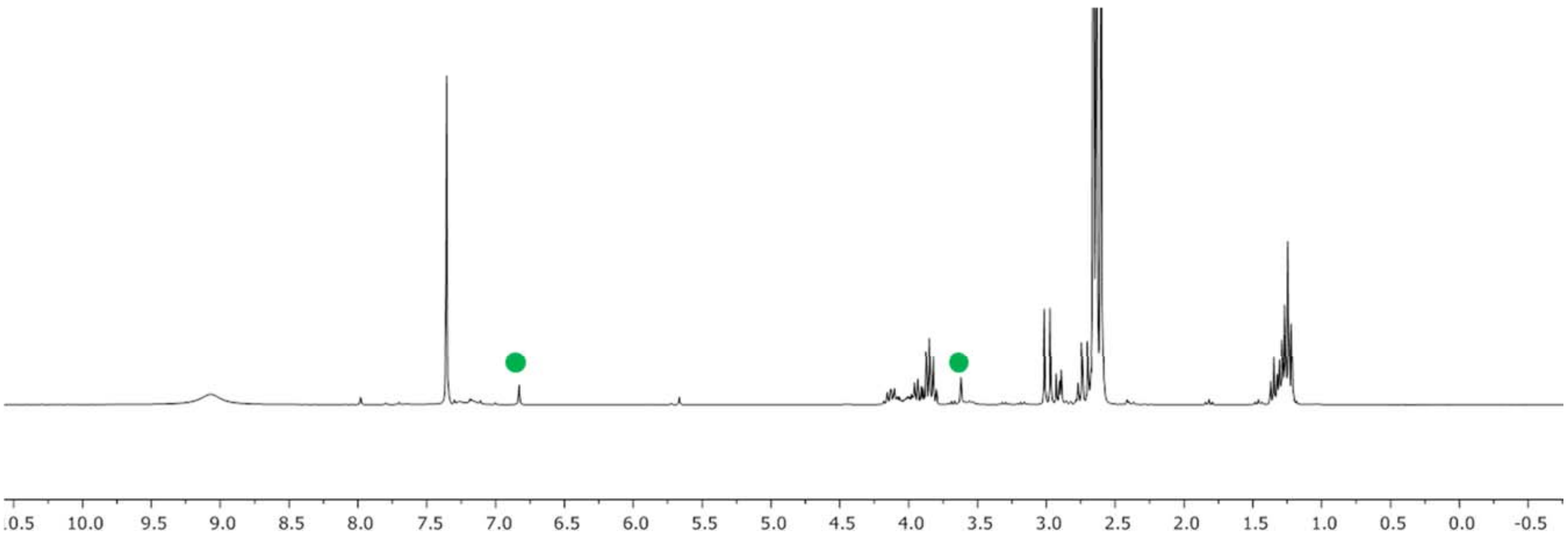

Supplementary Figure 12. ^{1}H NMR of crude mixture of one-pot reaction of H_2**L**$^{\mathbf{1}}$ with $SbCl_3$, I_2 and phosphoramide in $CDCl_3$. **1** is indicated by green circles.

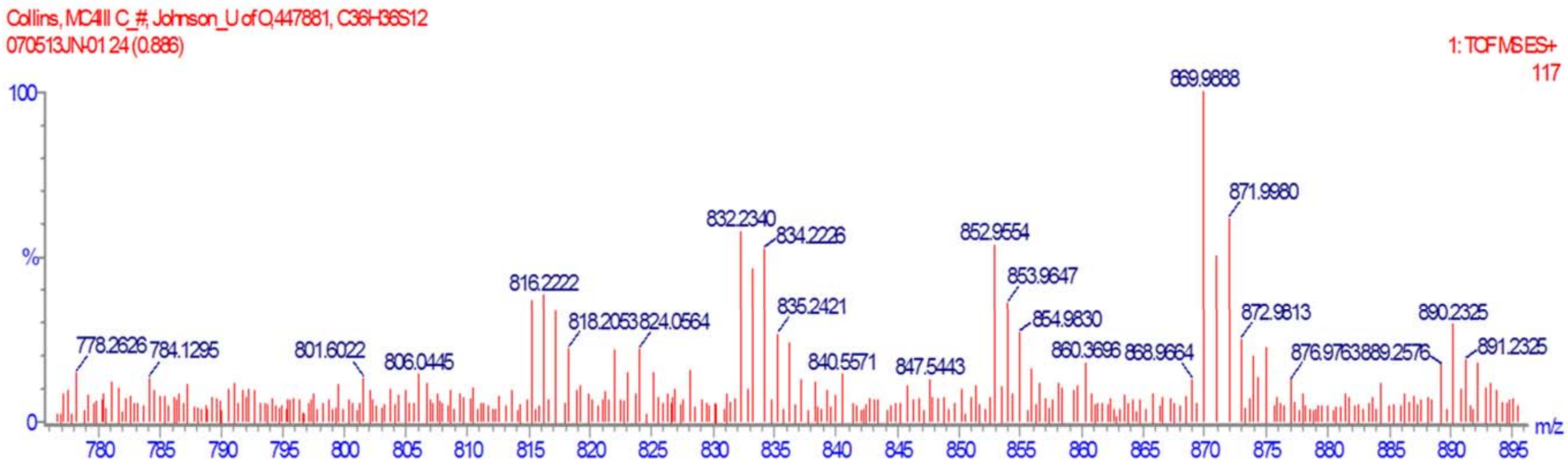

Supplementary Figure 13. L^3_4 HiRes Mass spectrometry.

Elemental Composition Report - HiRes

Single Mass Analysis

Tolerance = 100.0 PPM / DBE: min = -1.5, max = 50.0

Selected filters: None

Monoisotopic Mass, Even Electron Ions

24 formula(e) evaluated with 1 results within limits (all results (up to 1000) for each mass)

Elements Used:

C: 0-36 H: 0-37 Na: 0-1 S: 0-12

Minimum:				-1.5		
Maximum:		5.0	100.0	50.0		
Mass	Calc. Mass	mDa	PPM	DBE	i-FIT	Formula
852.9554	852.9544	1.0	1.2	18.5	2.8	C36 H37 S12

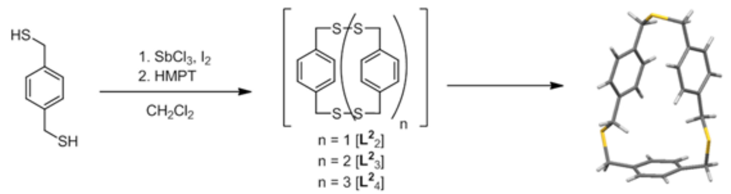

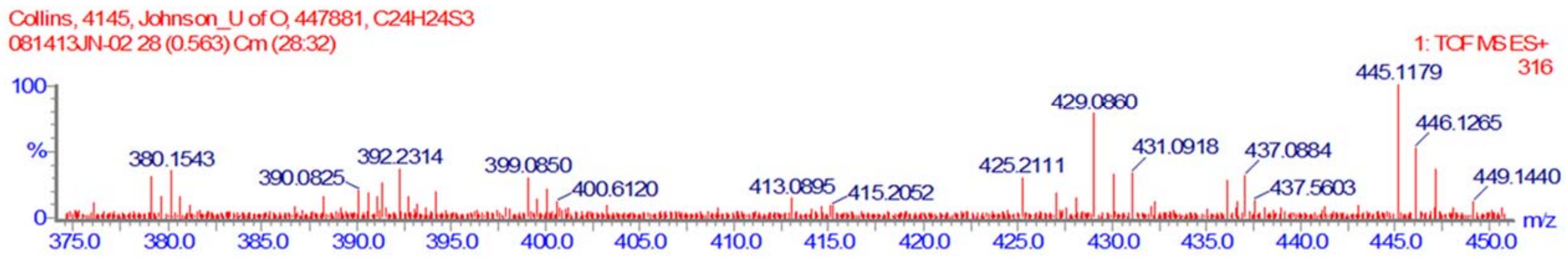

Supplementary Figure 14. 1- HiRes Mass spectrometry in crude reaction mixture.

Elemental Composition Report

Single Mass Analysis

Tolerance = 150.0 PPM / DBE: min = -1.5, max = 50.0

Selected filters: None

Monoisotopic Mass, Even Electron Ions

6 formula(e) evaluated with 1 results within limits (all results (up to 1000) for each mass)

Elements Used:

C: 0-24 H: 0-25 Na: 0-1 S: 0-3

Minimum:			-1.5
Maximum:	5.0	150.0	50.0

Mass	Calc. Mass	mDa	PPM	DBE	i-FIT	Formula
431.0918	431.0938	-2.0	-4.6	12.5	1.9	C24 H24 Na S3

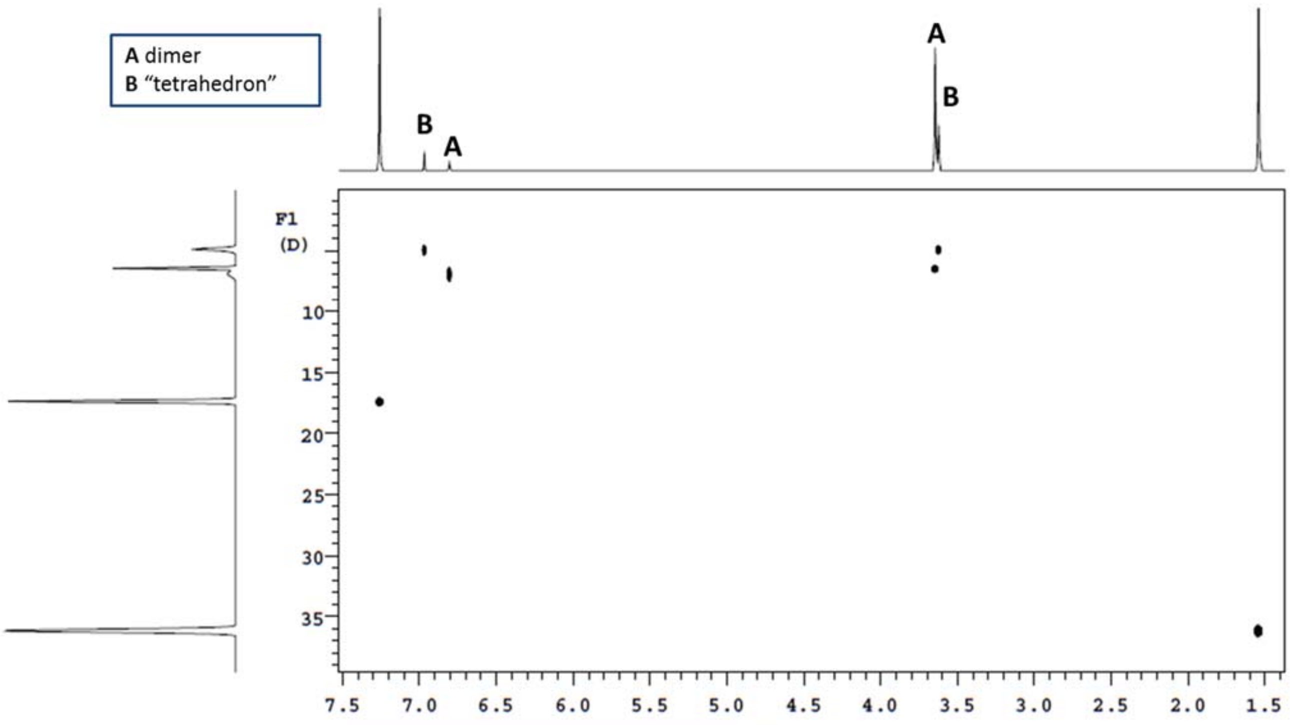

ppm	D (E-10)
6.9689	4.9379
6.8059	6.8993
3.6448	6.4897
3.6233	4.924

Supplementary Figure 15. 1H DOSY of crude reaction mixture of $\mathbf{L^3}_2$ (A; dimer) and $\mathbf{L^3}_4$ (B; tetrahedron) in crude reaction mixture in $CDCl_3$ – 500MHz.

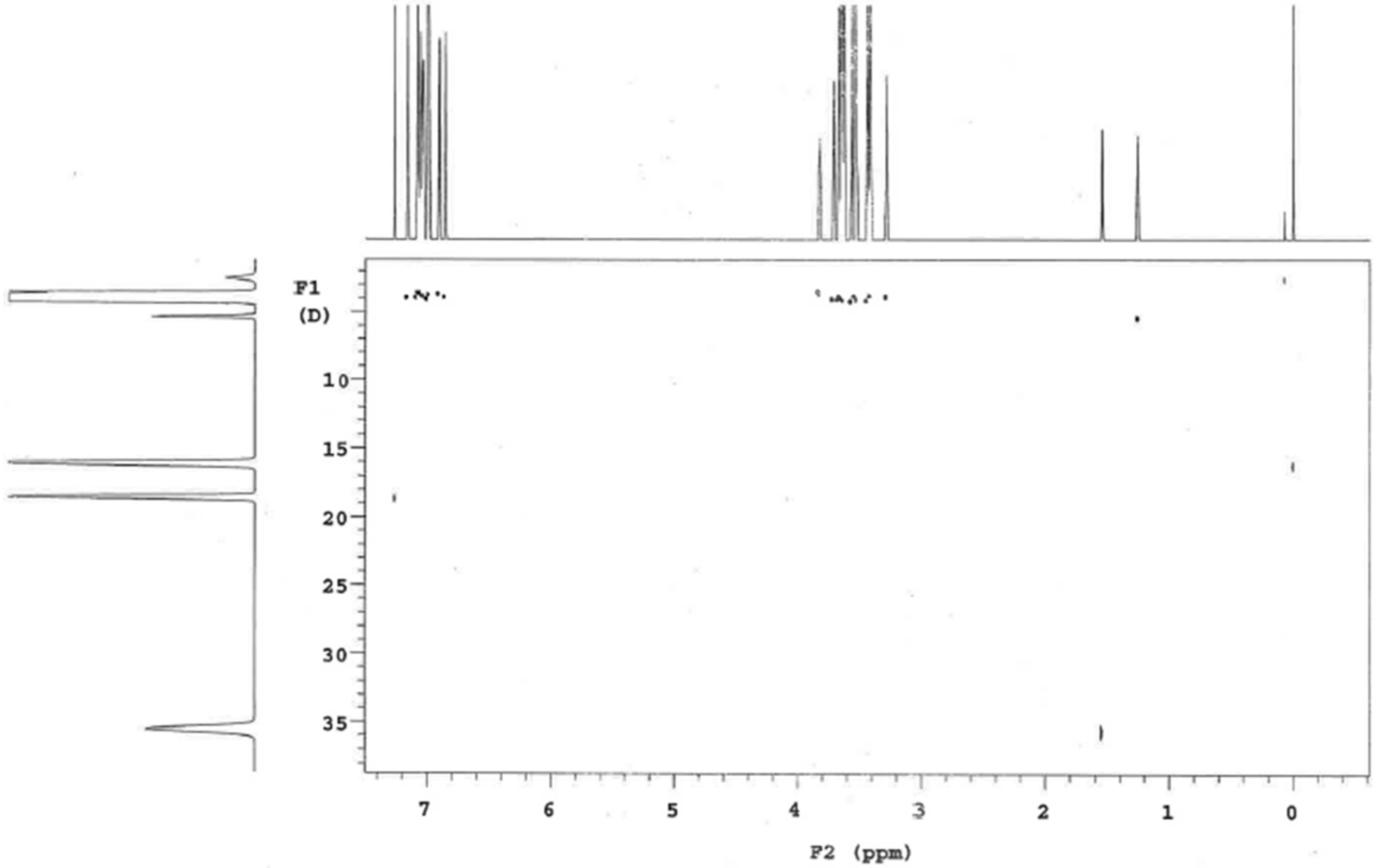

Supplementary Figure 16. ^{1}H DOSY of crude reaction mixture of an isolated species larger than $\mathbf{L^3}_4$ (perhaps a tetramer dimer or octamer) in $CDCl_3$ – 500 MHz (H_2O – 1.5 ppm; H grease – 1.25, 0.84-0.87 ppm).

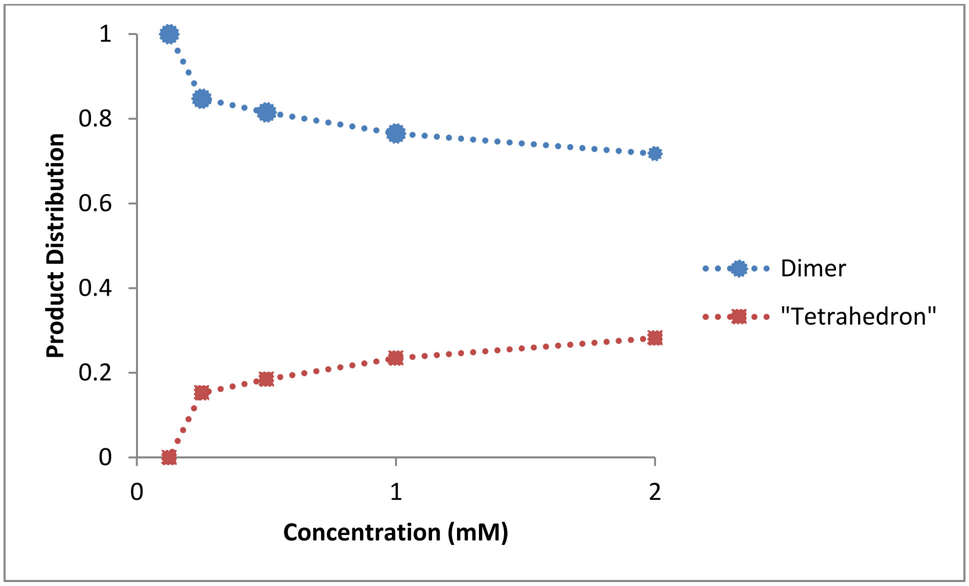

Supplementary Figure 17. Product distribution of [**L**$^3{}_2$] dimer and [**L**$^3{}_4$] tetrahedron as a function of starting dithiol concentration. Each reaction was run in $CDCl_3$ with stoichiometric amounts of antimony trichloride and iodine.

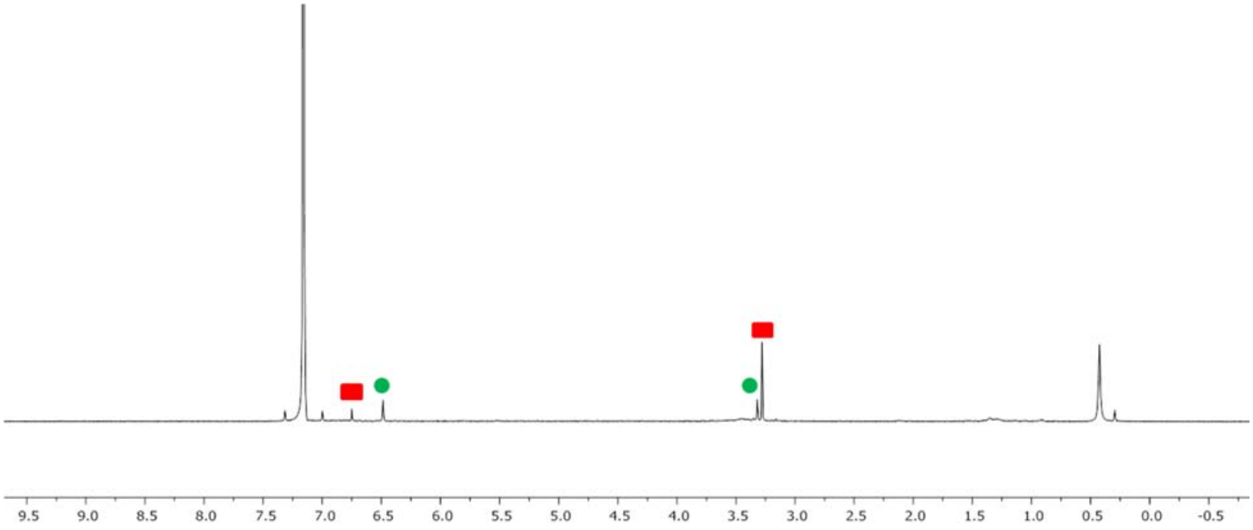

Supplementary Figure 18. NMR exhibiting normalized product distribution of [**L**$^3{}_2$] dimer (green circle) and [**L**$^3{}_4$] tetrahedron (red square) (39:61 ratio favoring tetrahedron [**L**$^3{}_4$]) in C_6D_6 – 500 MHz. To favor the formation of tetrahedron, the reaction was performed using the same conditions seen in Supplementary Figure 4, but using benzene as solvent instead of chloroform.

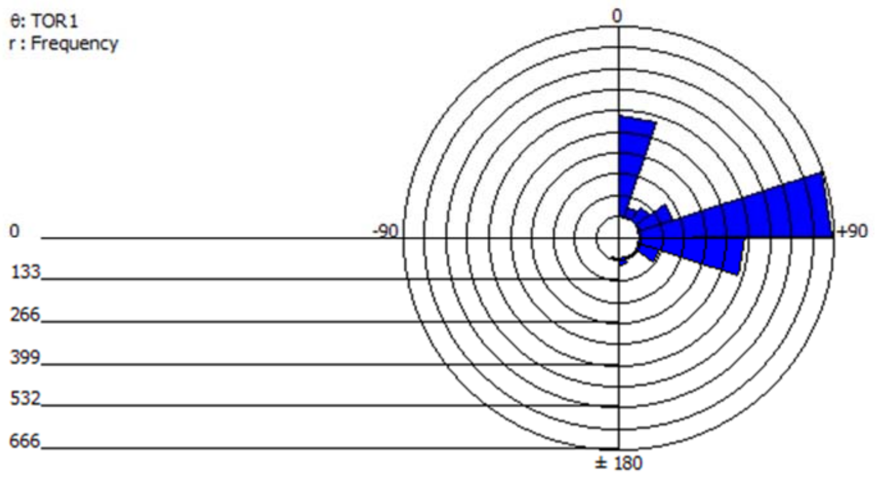

Supplementary Figure 19. Polar histogram of CSD search of disulfide bonds with dihedral angles between 0°-180° from a survey of the Cambridge Structural Database (CSD) for torsional angles of disulfide bonds[4]

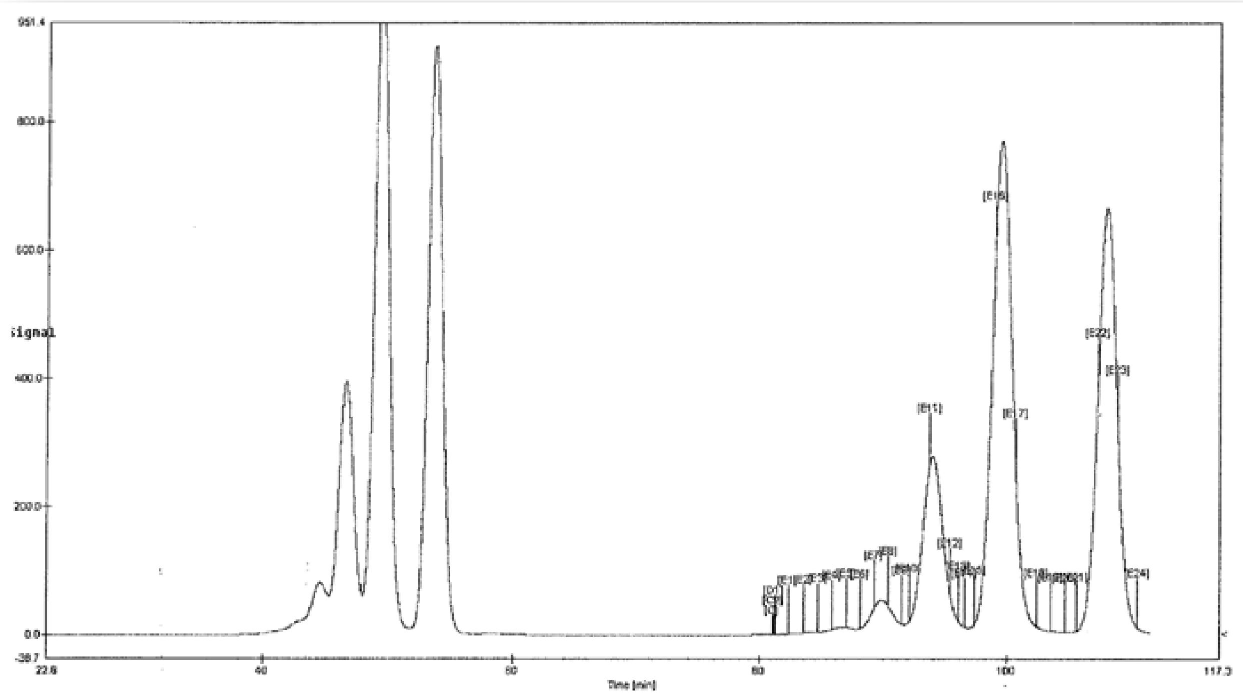

Supplementary Figure 20. Gel Permation Chromatogram of $\mathbf{L^2}_2$ (dimer), $\mathbf{L^2}_3$ (trimer), $\mathbf{L^2}_4$ (tetramer), $\mathbf{L^2}_5$ (pentamer) and $\mathbf{L^2}_6$ (hexamer) (peaks going from right to left). The mixture was purified in chloroform using a recycling HPLC.

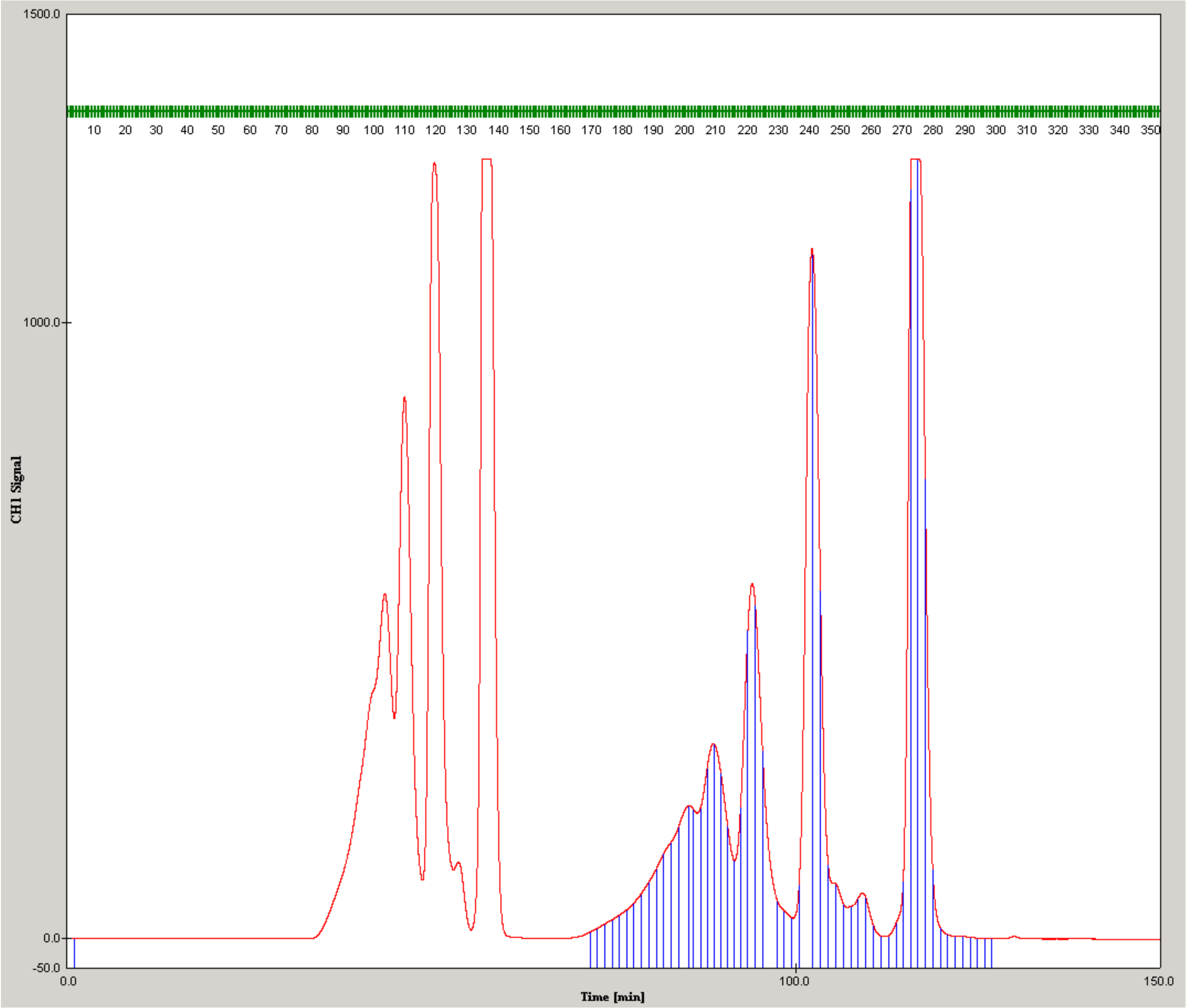

Supplementary Figure 21. Gel Permeation Chromatogram of $\mathbf{L^3}_2$ (dimer) and $\mathbf{L^3}_4$ (tetrahedron) (peaks going from right to left). The larger species has been isolated and reveals distinct diffusion coefficients suggesting a single, asymmetric species in solution, but remains uncharacterized structurally (see Supplementary Figure 16). The mixture was purified in chloroform using a recycling HPLC.

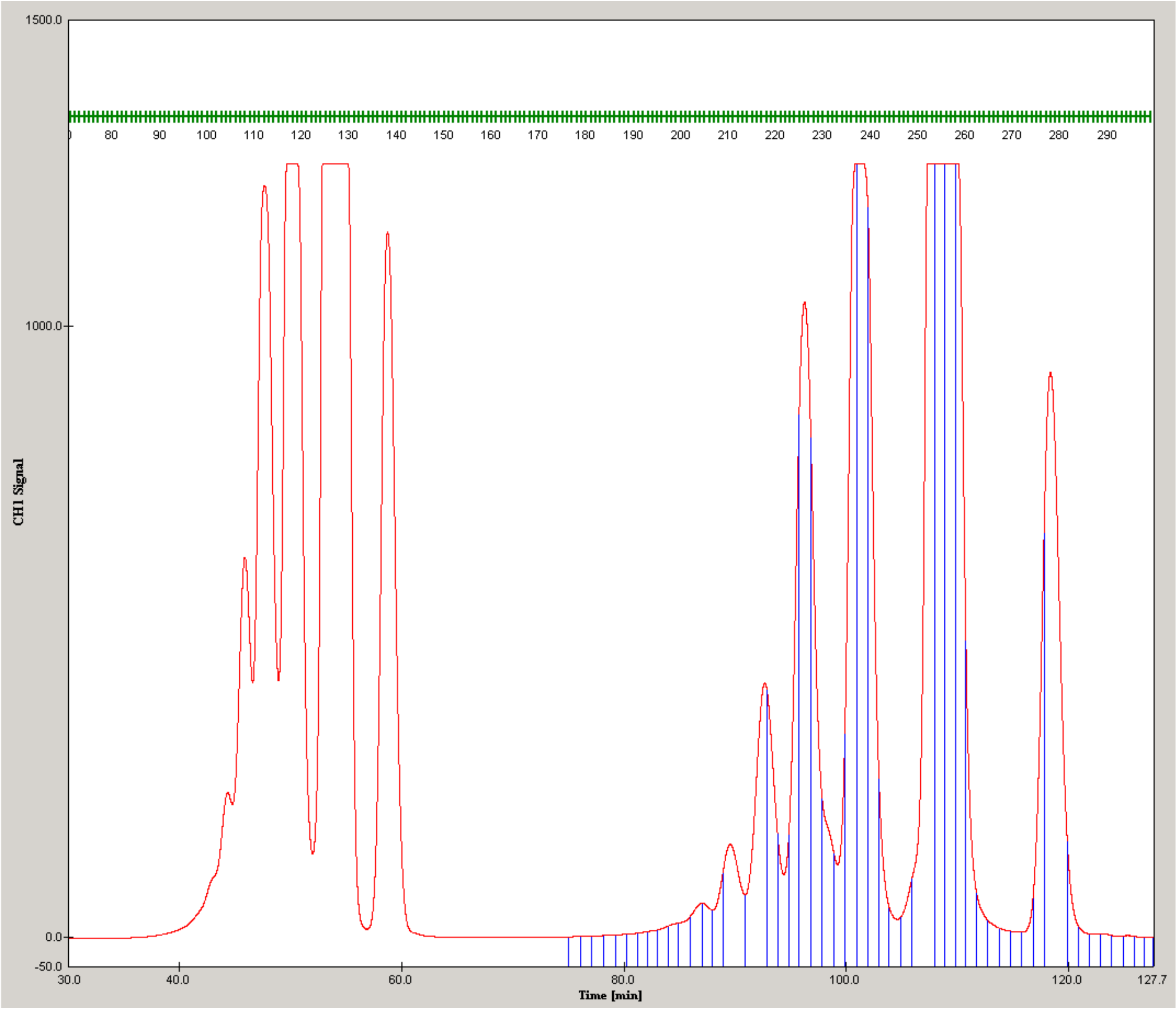

Supplementary Figure 22. Gel Permeation Chromatogram of $\mathbf{L^1}_2$ (dimer), $\mathbf{L^1}_3$ (trimer), $\mathbf{L^1}_4$ (tetramer), $\mathbf{L^1}_5$ (pentamer) and $\mathbf{L^1}_6$ (hexamer) (peaks going from right to left).The mixture was purified in $CDCl_3$ using a recycling HPLC.

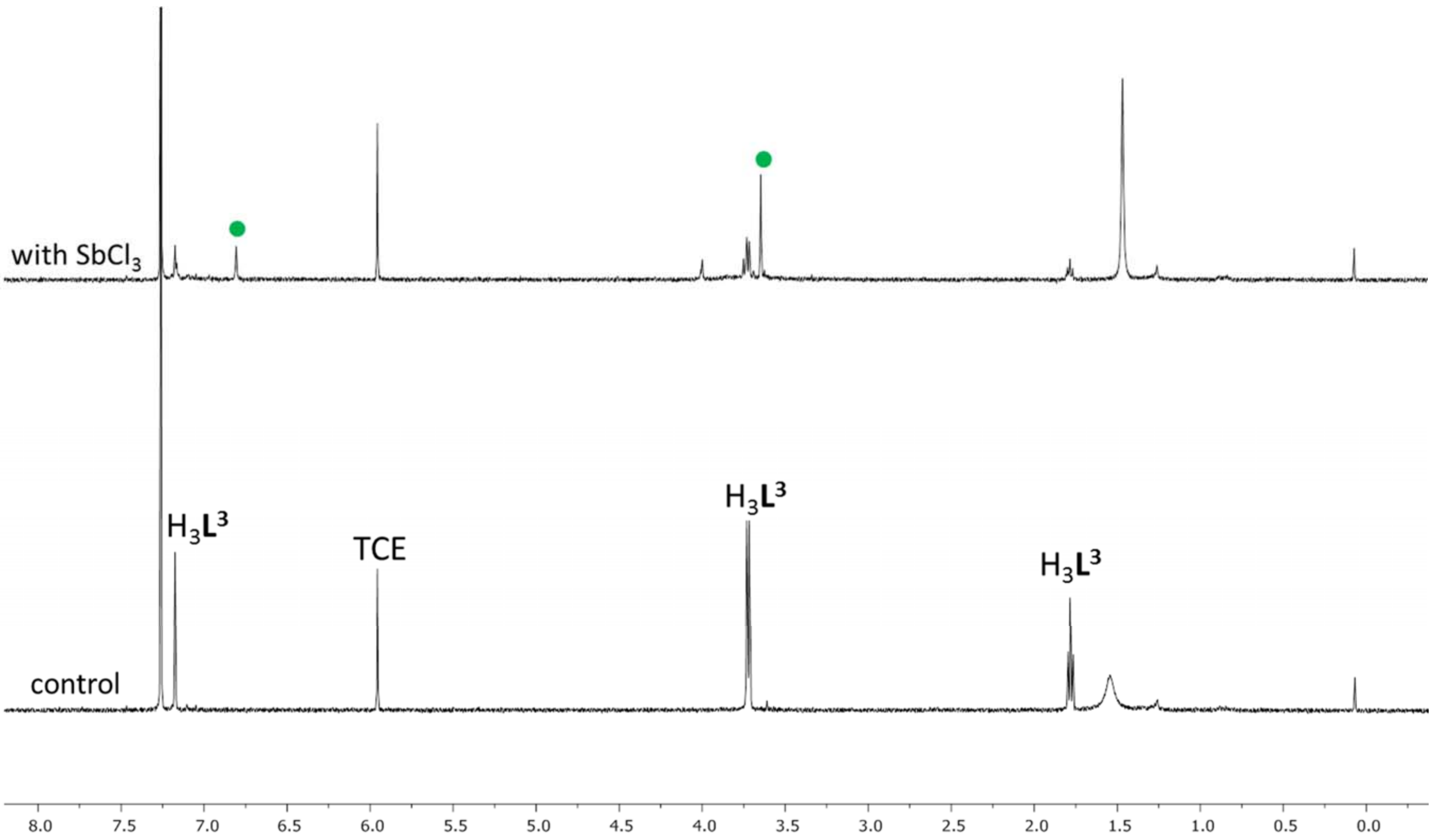

Supplementary Figure 23. Control reactions 1 min after addition of reagents: H_3**L**^{3} with I_2 (3/2 mmol) and $SbCl_3$ (0.8 mmol) in $CDCl_3$ with a 1,1,2,2-tetrachloroethane (TCE) standard – top; H_3**L**^{3} with I_2 (3/2 mmol) only in $CDCl_3$ with TCE standard – bottom. **L**$^3{}_2$ (dimer) is indicated by green circles.

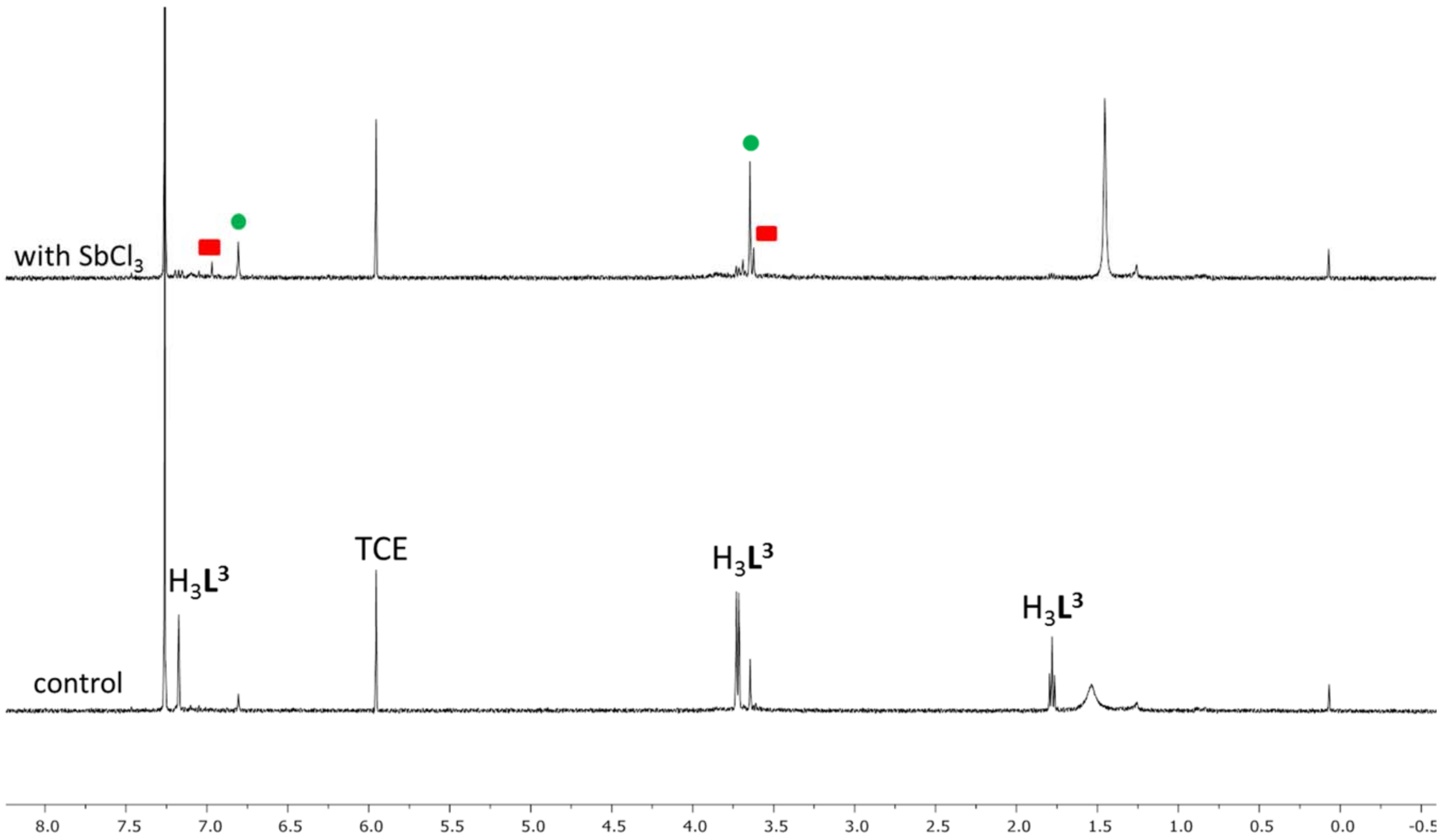

Supplementary Figure 24. The same control reactions (Supplementary Figure 23) 1 h after addition of reagents: $H_3\mathbf{L}^3$ with I_2 (3/2 mmol) and $SbCl_3$ (0.8 mmol) in $CDCl_3$ with a 1,1,2,2-tetrachloroethane (TCE) standard – top; $H_3\mathbf{L}^3$ with I_2 (3/2 mmol) only in $CDCl_3$ with TCE standard – bottom. $\mathbf{L}^3{}_2$ (dimer) is indicated by green circles and $\mathbf{L}^3{}_4$ (tetrahedron) by red squares.

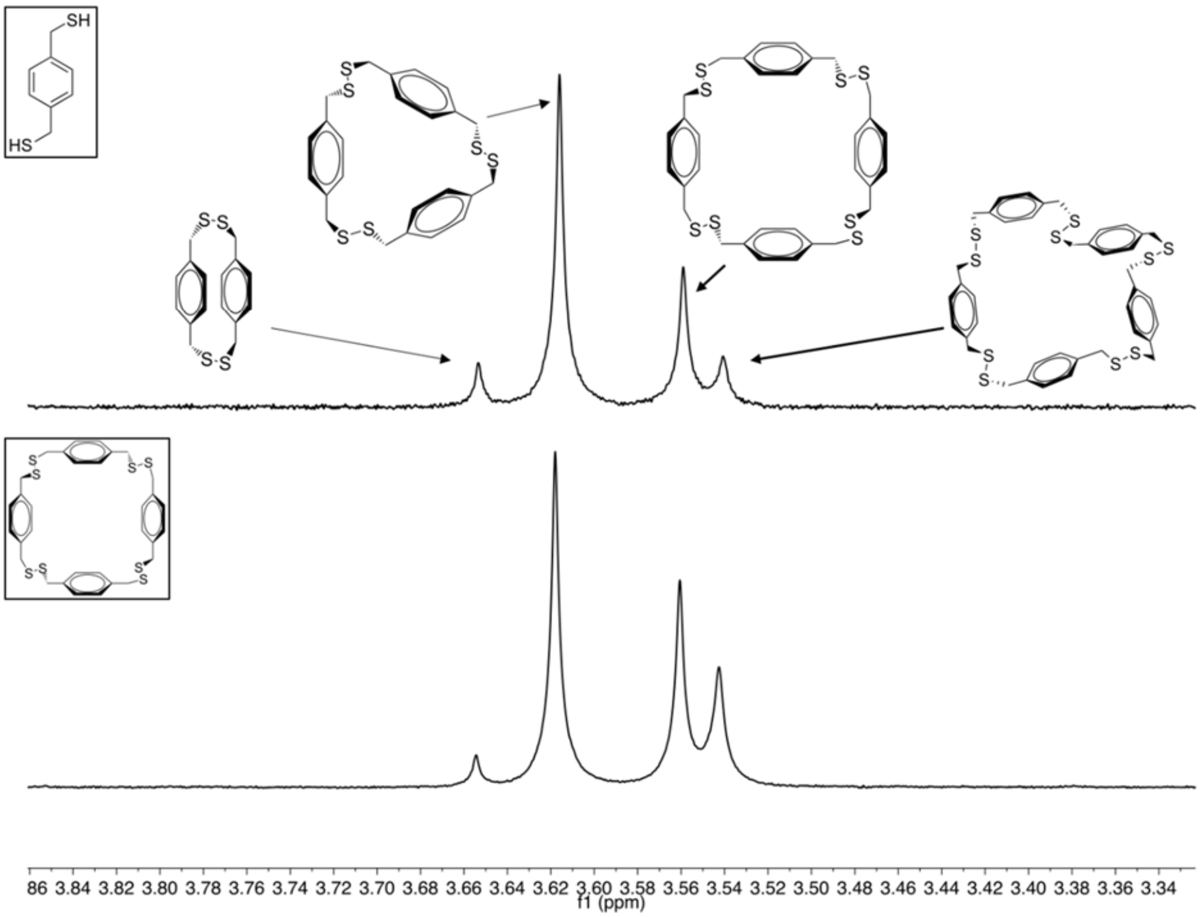

Supplementary Figure 25. (top) ^{1}H NMR spectra showing the distribution of macrocyclic disulfides formed upon treatment of ligand $H_2\mathbf{L}^1$ (1 equiv), $SbCl_3$ (1.0 equiv), and $\mathbf{I}_2$ (2.1 equiv) at 6.2 mM, showing a mixture of dimer, trimer, tetramer, and pentamer. (bottom) The same distribution of macrocyclic disulfides is formed upon treatment of tetrameric disulfide macrocycle $[\mathbf{L}^1_4]$ (1 equiv), $SbCl_3$ (1.1 equiv), and $\mathbf{I}_2$ (2.4 equiv) at 5.5 mM, consistent with the self-assembly reaction being under thermodynamic control.

Supplementary Discussion

Crystal Structure CH_2 (methylene) Deviation from Planarity for $L^3{}_2$

There are two symmetrically independent molecules
The average plane is C(2)-C(7). The deviation for C1 is 0.XXX(XX)
"+" and "-" show direction of the deviation.

Least-squares planes (x,y,z in crystal coordinates) and deviations from them
(* indicates atom used to define plane)

- 7.3457 (0.0059) x + 0.2961 (0.0455) y + 7.6103 (0.0375) z = 5.9834 (0.0489)

*-0.0029 (0.0045) C2
*-0.0005 (0.0046) C3
*-0.0009 (0.0042) C4
* 0.0056 (0.0041) C5
*-0.0090 (0.0042) C6
* 0.0076 (0.0044) C7
-0.1233 (0.0118) C1
-0.1490 (0.0114) C8_a
-0.1802 (0.0114) C9_a

Rms deviation of fitted atoms = 0.0055

7.3907 (0.0053) x - 0.2342 (0.0462) y + 0.2122 (0.0421) z = 1.8493 (0.0339)

Angle to previous plane (with approximate esd) = 30.365 (0.131)

*-0.0006 (0.0045) C2'
*-0.0080 (0.0044) C3'
* 0.0092 (0.0043) C4'
*-0.0019 (0.0043) C5'
*-0.0066 (0.0044) C6'
* 0.0079 (0.0047) C7'
0.1258 (0.0113) C1'
0.1855 (0.0110) C8'_a
0.1297 (0.0114) C9'_a

Rms deviation of fitted atoms = 0.0066

Supplementary Methods

General Information

^{1}H NMR spectra were measured using Varian INOVA-300, 500, and 600 spectrometers and ^{13}C Varian INOVA-600 spectrometer in $CDCl_3$ CD_2Cl_2, C_6D_6 and TCE-d_2. Spectra were referenced using the residual solvent resonances as internal standards and reported in ppm. The DOSY experiments were performed using the gradient stimulated echo with spinlock and convection compensation (DgsteSL_cc) pulse sequences. All Varian software standard default settings were kept for DOSY unless otherwise stated. The diffusion delay was increased to 100 ms, the number of increments was increased to 30, and the highest gradient value was set to 25,000. Single crystal X-ray diffraction studies were performed on a

Bruker SMART10.1055/s-0030-1259306 APEX diffractometer. Commercially available reagents were used as received. The reported yields are for isolated sample. *Caution: Antimony compounds are toxic and should be handled with care!* (This accounts for the small scale of the reactions reported herein.) The preparation of 1,4-bis(thiomethy)benzene (H_2**L**^{1}), 1,4-dimethoxy-2,5-bis(mercaptomethyl)benzene (H_2**L**^{2}) and 2,3,12,13-Tetrathia[4.4]paracyclophane (**L**$^1{}_2$), 2,3,12,13,22, 23-hexathia[4.4.4]paracyclophane (**L**$^1{}_3$), and 2,3,12,13, 22, 23, 32, 33-octathia[4.4.4.4]paracyclophane (**L**$^1{}_4$) was previously reported.[1] Synthesis and characterization of 1,3,5-tris(thiomethyl)benzene (H_3**L**^{3}) was also previously reported.[2] Pnictogen-activated iodine oxidation to give discrete disulfide complexes occurs instantaneously. The reactions disclosed are reacted overnight in order to allow for greater product conversion to the larger species *ie.* pentamers, hexamers and tetrahedra. The scale-up reaction (**3**) was performed reproducibly, although it is un-optimized. Most of the loss of product arises from the purification of the thioether. Further optimization will require tuning the solvent system and concentration to minimize oxidation and improve isolation of the final thioether during the desulfurization step; these conditions will continue to be optimized for various cyclophanes and will be reported in due course. These results suggest that the preparation of thioether cyclophanes is general and scalable using standard laboratory purification techniques.

Ligand Synthesis

Synthesis of 1,3,5-tris(thiomethyl)benzene (H_3L^3). 1,3,5-tris(bromomethyl)benzene (5.0 g, 14 mmol) and thiourea (6.40 g, 84.1 mmol) were dissolved in acetone (400 mL) and stirred for 16 h at 63 °C. The thiouronium salt was filtered, placed in a 1L round bottom flask and purged with N_2. Degassed 3 M NaOH (250 mL) was cannulated into the flask and the solution was stirred for 2 h at 80 °C. The reaction mixture was removed from heat and degassed 9 M HCl (200mL) was cannulated into the flask alternating with degassed $CHCl_3$ (150 mL) until pH 2. The chloroform fraction was separated from the aqueous and more product was extracted with 3X with $CHCl_3$ and washed with brine. The organic solution was dried with Na_2SO_4, and filtered. The filtrate was concentrated to give a yellow oil (84%). ^{1}H NMR (300 MHz, $CDCl_3$): δ 7.17 (s, 3H, C_6H_3), 3.73 (d, 6H, CH_2, J = 7.6 Hz), 1.79 (t, 3H, SH, J = 7.6 Hz)

Disulfide Synthesis

Synthesis of 2,3,12,13, 22, 23, 32, 33, 42, 43-decathia[4.4.4.4.4]paracyclophane ($L^1{}_5$) and 2,3,12,13, 22, 23,32,33,42,43,52,53-dodecathia[4.4.4.4.4.4]paracyclophane ($L^1{}_6$).[1] H_2**L**^{1} (89 mg, 0.52 mmol) was added to $CHCl_3$ (50 mL) in a flask equipped with a stir bar. A second flask was charged with I_2 (264 mg, 1.04 mmol) and $SbCl_3$ (59 mg, 0.26 mmol) in 50 mL $CHCl_3$. The solution of I_2 and $SbCl_3$ was then added slowly to the solution of H_3**L**^{1} while stirring. The dark purple solution was then allowed to stir at ambient temperature for 16 h. The desired disulfide complexes were obtained by quenching with sodium sulfite followed with washing the chloroform mixture with deionized water (2X). The solution was dried with Na_2SO_4, filtered and concentrated under reduced pressure to afford a white powder. The powder was then redissolved in 3 mL of chloroform and purified by GPC (84% combined yield: 9% dimer; 45% trimer, 20% tetramer, 7% pentamer, 3% hexamer). X-ray quality crystals were grown by solvent evaporation from chloroform.

Synthesis of 6,9,16,19-tetramethoxy-2,3,12,13-tetrathia[4.4]paracyclophane, 6,9,16,19,26,29-hexamethoxy-2,3,12,13, 22,23-hexathia[4.4.4]paracyclophane, 6,9,16,19,26,29,36,39-octamethoxy-2,3,12,13, 22,23, 32,33-octathia[4.4.4.4]paracyclophane, 6,9,16,19,26,29,36,39, 46, 49-decamethoxy-2,3,12,13, 22,23, 32,33, 42,43-decathia[4.4.4.4.4]paracyclophane, 6,9,16,19,26,29,36,39, 46, 49, 56, 59-dodecamethoxy-2,3,12,13, 22,23, 32,33, 42,43,52,53-dodecathia[4.4.4.4.4.4]paracyclophane, ($L^2{}_2$, $L^2{}_3$, $L^2{}_4$, $L^2{}_5$, and $L^2{}_6$). Under an atmosphere of ambient air, H_2**L**^{2} (25 mg, 0.11 mmol) was added to $CHCl_3$ (25 mL) in a flask equipped with a stir bar. A second flask was charged with I_2 (56 mg, 0.22 mmol) and $SbCl_3$ (13 mg, 0.055 mmol) in 25 mL $CHCl_3$. The solution of I_2 and $SbCl_3$ was then added slowly to

the solution of H_3**L**^{2} while stirring. The dark purple solution was then allowed to stir at ambient temperature for 16 h. The desired disulfide complexes were obtained by quenching with sodium sulfite followed with washing the chloroform mixture with deionized water (2X). The solution was dried with Na_2SO_4, filtered and concentrated under reduced pressure to afford a white powder. The powder was then redissolved in 3 mL of chloroform and purified by GPC (93% combined yield: 37% dimer; 35% trimer, 13% tetramer, 4% pentamer, 4% hexamer). ^{1}H NMR ($CDCl_3$) of (**L**$^{2}_{2}$): δ = 6.42 (bs, 4H, C_6H_2), 3.76 (s, 12H, CH_3), 3.85 (bs, 4H, CH_2), 3.23 (bs, 4H, CH_2); ^{13}C{^{1}H} NMR (150 MHz, $CDCl_3$): δ = 150.9, 150.3, 126.2, 126.1, 114.0, 113.5, 56.4, 56.0, 39.4, 38.7 ppm; (**L**$^{2}_{3}$): δ = 6.53 (s, 6H, C_6H_2), 3.78 (s, 18H, CH_3), 3.59 (s, 12H, CH_2); ^{13}C{^{1}H} NMR (150 MHz, $CDCl_3$): δ = 150.3, 126.2, 113.4, 56.0, 39.4 ppm; (**L**$^{2}_{4}$): δ = 6.65 (s, 8H, C_6H_2), 3.76 (s, 24H, CH_3), 3.62 (s, 16H, CH_2); ^{13}C{^{1}H} NMR (150 MHz, $CDCl_3$): δ = 150.9, 126.1, 114.0, 56.4, 38.7 ppm; (**L**$^{2}_{5}$): δ = 6.64 (s, 10H, C_6H_2), 3.75 (s, 30H, CH_3), 3.65 (s, 20H, CH_2), ^{13}C{^{1}H} NMR (150 MHz, $CDCl_3$): δ = 151.0, 126.0, 114.1, 56.4, 38.6 ppm; (**L**$^{2}_{6}$): δ = 6.66 (s, 12H, C_6H_2), 3.76 (s, 36H, CH_3), 3.68 (s, 24H, CH_2).

Synthesis of disulfide 2,3,12,13,22,23,33,34,42,43,46,47-dodecathia[4.4.4.4.4.4](1,3,5)benzenophane and 2,3,12,13,22,23-hexathia[4.4.4](1,3,5)benzenophane (dodecathiatetrahedrophane, L$^{3}_{4}$ and dimer, L$^{3}_{2}$). Under an atmosphere of ambient air, H_3**L**^{3} (90 mg, 0.42 mmol) was added to $CHCl_3$ (100 mL) in a flask equipped with a stir bar. A second flask was charged with I_2 (317 mg, 1.25 mmol) and $SbCl_3$ (237 mg, 1.04 mmol) in 100 mL $CHCl_3$. The solution of I_2 and $SbCl_3$ was then added slowly to the solution of H_3**L**^{3} while stirring. The resulting mixture was then allowed to stir at ambient temperature for 16 h to afford a clear, dark purple solution. The desired disulfide complexes were obtained by quenching with sat. sodium sulfite followed with washing the chloroform mixture with deionized water (2X). The solution was dried with Na_2SO_4, filtered and concentrated to under reduced pressure to afford a white powder. The powder was then redissolved in 3 mL of chloroform and purified by GPC (98% combined yield: 69% dimer; 29% tetramer). ^{1}H NMR (600MHz, $CDCl_3$) of (**L**$^{3}_{4}$): δ = 6.97 (s, 12H, C_6H_3) 3.62 (s, 24H, CH_2); ^{13}C{^{1}H} NMR (150 MHz, $CDCl_3$): δ = 138.7, 129.1, 44.9 ppm. (**L**$^{3}_{2}$) ^{1}H NMR (600 MHz, $CDCl_3$): δ = 6.81 (s, 6H, C_6H_3), 3.64 (s, 12H, CH_2). ^{13}C{^{1}H} NMR (150 MHz, $CDCl_3$): δ = 138.7, 129.0, 43.7 ppm.

Synthesis of thiacyclophanes

Synthesis of 2,11,20-trithia[3.3.3]paracyclophane (1). Two acid-washed, oven-dried NMR tubes were charged with **L**$^{1}_{3}$ (32 mg, 0.064 mmol; 16 mg in per tube) in 4 mL chloroform dried with 4A molecular sieves (2 mL per tube). Under a cone of nitrogen, HMPT (38 µL, 0.21 mmol; 13 µL per tube) was added to NMR tubes and the tube was inverted gently several times to mix. The reaction was allowed to sit at ambient temperature for 3 h. The solution was then concentrated down and the crude solid was sonicated with 30 mL of deionized water giving a cloudy white solution. The solid was separated from its aqueous counterpart by centrifugation, and washed a second time with fresh deionized water. The white pellet was then redissolved in chloroform and passed through a short silica plug (82% isolated yield). ^{1}H NMR (300 MHz, $CDCl_3$) of (**L**$^{3}_{4}$): δ = 6.84 (s, 6H, C_6H_4) 3.62 (s, 12H, CH_2); ^{13}C{^{1}H} NMR (150 MHz, $CDCl_3$): δ = 138.7, 129.1, 44.9 ppm.

Synthesis of 5,8,14,17,23,26-hexamethoxy-2,11,20-trithia[3.3.3]paracyclophane (2). L^2_3 (13 mg, 0.018 mmol) was dissolved in 1 mL of CD_2Cl_2 and transferred to an oven-dried NMR tube. HMPT (13 µL, 0.074 mmol) was added to the NMR tube, and the tube was shaken vigorously. Reaction is observed to be complete in 4 h by ^{1}H NMR giving the desired thioether trimer (72% isolated yield after centrifuge purification). ^{1}H NMR (500 MHz, CD_2Cl_2): δ = 6.53 (s, 6H, C_6H_2), 3.70 (s, 12H, CH_2), 3.51(s, 18H, CH_3). ^{13}C{^{1}H} NMR (125 MHz, CD_2Cl_2): δ = 151.1, 127.0, 113.0, 56.2, 31.1 ppm.

Synthesis of 2,11,20-trithia[3.3.3](1,3,5)cyclophane[3] (3). L^3_2 (5.0 mg, 0.011 mmol) was dissolved in 1 mL of degassed $CDCl_3$ (4A molecular sieve dried) and transferred to an oven-dried NMR tube. HMPT (7.0 µL, 0.44 mmol) was added to the NMR tube, and the tube was shaken vigorously. Reaction is observed to be complete in 2 h by 1H NMR at 100% conversion to desired thiacyclophane dimer (92% isolated yield after centrifuge purification). ^{1}H NMR (300 MHz, $CDCl_3$): δ = 6.90 (s, 6H, C_6H_3), 3.85 (s, 12H, CH_2).

Scaled-up synthesis of 2,11,20-trithia[3.3.3](1,3,5)cyclophane (3). Under an atmosphere of ambient air, H_3**L**^{3} (0.480 g, 2.20 mmol) was added to $CHCl_3$ (500 mL) in a flask equipped with a stir bar. A second flask was charged with I_2 (2.300 g, 9.06 mmol) and $SbCl_3$ (1.15 g, 5.04 mmol) in 150 mL $CHCl_3$. The solution of I_2 and $SbCl_3$ was then added slowly to the solution of H_3**L**^{3} while stirring. The resulting mixture was then allowed to stir at ambient temperature for 5 h to afford a clear, dark purple solution. The desired disulfide complexes were obtained by quenching with sat. sodium sulfite followed with washing the chloroform mixture with deionized water (2X). The solution was dried with Na_2SO_4, filtered, and concentrated under reduced pressure to afford a white powder. A ^{1}H NMR spectrum of the crude powder indicated an 83:17 distribution of dimer:tetramer. The crude powder was then dissolved in 100 mL of degassed $CHCl_3$ (4Å molecular sieve dried) and transferred to an oven-dried round-bottomed flask under a nitrogen atmosphere. HMPT (2.62 mL, 14.4 mmol) was added to the flask, and the flask was shaken vigorously for 12 h. The reaction was observed to be complete with 100% conversion to the desired thiacyclophane mixture of dimer and tetramer by ^{1}H NMR. Excess phosphine was removed in vacuo and the powder residue was purified by column chromatography (80:20; $CHCl_3$:hexanes) to yield 113 mg of dimer (L^3_2) (31% isolated yield over two steps).

Synthesis of 2,11,20,29,38,41-hexathia[3.3.3](1,3,5)benzenophane (5). 10 mL of $CDCl_3$ dried under 4A molecular sieves was added to $\mathbf{L^3}_4$ (22 mg, 0.026 mmol) in an acid-washed, oven-dried glass vial. The vial was sonicated until the solution was evenly dispersed as a cloudy white mixture. HMPT (28 μL, 0.16 mmol) was added to the vial in which the solution alters from cloudy in appearance to clear and colorless within 15 minutes. The reaction was unmoved at ambient temperature for 4 h then concentrated down. The white solid was sonicated with deionized water and then transferred to a centrifuge tube. To remove any remaining HMPA, the product is washed 3X with deionized water by centrifugation: the solid is dispersed in water and centrifuged down. The aqueous supernatant is pipetted off the product pellet. This centrifugation wash was repeated two more times to thoroughly remove any residual water-soluble HMPA. The white solid is then dissolved in chloroform, dried with Na_2SO_4 and concentrated to give the final crystalline solid (94.5% isolated yield). (500 MHz, $CDCl_3$): δ = 6.83 (s, 12H, C_6H_3), 3.49 (s, 24H, CH_2). $^{13}C\{^1H\}$ NMR (150 MHz, $CDCl_3$): δ = 138.2, 127.8, 36.8 ppm.

Supplementary References

1. Collins, M. S. *et al.* Pnictogen-directed synthesis of discrete disulfide macrocycles. *Chem. Commun.* **49,** 6599–601 (2013).

2. Houk, J. & Whitesides, G. M. Structure-reactivity relations for thiol-disulfide interchange. *J. Am. Chem. Soc.* **109,** 6825–6836 (1987).

3. Boekelheide, V. & Hollins, R. A. Syntheses of novel tris-bridged cyclophanes. [2.2.2](1,3,5)cyclophane-1,9,17-triene. *J. Am. Chem. Soc.* **92,** 3512–3513 (1970).
